# Research Capacity Strengthening in Low and Middle Income Countries – An Evaluation of the WHO/TDR Career Development Fellowship Programme

**DOI:** 10.1371/journal.pntd.0004631

**Published:** 2016-05-25

**Authors:** Michael Käser, Christine Maure, Beatrice M. M. Halpaap, Mahnaz Vahedi, Sara Yamaka, Pascal Launois, Núria Casamitjana

**Affiliations:** 1 Swiss Tropical and Public Health Institute, Basel, Switzerland; 2 University of Basel, Basel, Switzerland; 3 World Health Organization (WHO), Geneva, Switzerland; 4 Special Programme for Research and Training in Tropical Diseases (WHO/TDR), Geneva, Switzerland; 5 ISGlobal, Barcelona Institute for Global Health, Hospital Clínic—Universitat de Barcelona, Barcelona, Spain; KEMRI, KENYA

## Abstract

Between August 2012 and April 2013 the Career Development Fellowship programme of the Special Programme for Research and Training in Tropical Diseases (World Health Organization) underwent an external evaluation to assess its past performance and determine recommendations for future programme development and continuous performance improvement. The programme provides a year-long training experience for qualified researchers from low and middle income countries at pharmaceutical companies or product development partnerships. Independent evaluators from the Swiss Tropical and Public Health Institute and the Barcelona Institute for Global Health used a results-based methodology to review the programme. Data were gathered through document review, surveys, and interviews with a range of programme participants. The final evaluation report found the Career Development Fellowship to be relevant to organizers’ and programme objectives, efficient in its operations, and effective in its training scheme, which was found to address needs and gaps for both fellows and their home institutions. Evaluators found that the programme has the potential for impact and sustainability beyond the programme period, especially with the successful reintegration of fellows into their home institutions, through which newly-developed skills can be shared at the institutional level. Recommendations included the development of a scheme to support the re-integration of fellows into their home institutions post-fellowship and to seek partnerships to facilitate the scaling-up of the programme. The impact of the Professional Membership Scheme, an online professional development tool launched through the programme, beyond the scope of the Career Development Fellowship programme itself to other applications, has been identified as a positive unintended outcome. The results of this evaluation may be of interest for other efforts in the field of research capacity strengthening in LMICs or, generally, to other professional development schemes of a similar structure.

## Author Summary

The asymmetry of research training between high and low and middle income countries (LMICs) and the resulting need for research capacity strengthening (RCS) in under-resourced regions has long been established. In 1999, the World Health Organization (WHO), through the Special Programme for Research and Training in Tropical Diseases (TDR), launched the Career Development Fellowship (CDF) programme, in which LMICs’ candidates spend one year in a pharmaceutical company to be trained in product development and clinical research and then return to their home institution. Between August 2012 and April 2013, the programme underwent an independent external evaluation from the perspectives of relevance, effectiveness, efficiency, and impact, and sustainability. In addition to analysis of data and documentation, programme managers, current and former fellows, home and host institutions participated in the evaluation through surveys and interviews. In the current article, the external evaluators, along with CDF programme managers, discuss the results of the evaluation in the broader context of individual and institutional RCS, underlining CDF successes, including transmitting essential skills and networks, and addressing the challenge and fundamental importance of integrating fellows, home and host institutions into an on-going relationship that solidifies and amplifies the benefits of the one-year experience.

## Introduction

The gap in research capacity strengthening (RCS) has been widely recognized as a major stumbling block for development in low and middle-income countries (LMICs) and is manifested in the absence of trained and experienced candidates with skills, knowledge, networks, and confidence to build careers in research [1–3]. Both cause for and consequence of this deficit in RCS is the phenomenon called ‘brain drain’: young skilled candidates for a research career go abroad to find better research environments and working conditions. Hence, this continuing exodus to industrialized countries results in reduced numbers of trained scientists in LMICs [4]. This is observable also in health-related research areas where LMICs continue to lag behind industrialized countries although in these very places key improvements in public health stand to be achieved through evidence-based clinical research.

Founded to promote high-quality clinical research in LMICs, the Career Development Fellowship (CDF) programme began in 1999 as a partnership between the World Health Organization Special Programme for Research and Training in Tropical Diseases (WHO/TDR) and one pharmaceutical company [5]. During the initial phase of the programme (1999–2009), WHO/TDR awarded one fellowship per year to nine candidates originating from seven LMICs. The fellows were all placed with GlaxoSmithKline Biologicals, the Belgium-based division of the global vaccine and pharmaceutical research, development, and production company. This early phase (Phase I) assisted WHO/TDR to identify and develop appropriate procedures and mechanisms to support and administer the fellowship programme. In the second period (Phase II—2008–2013) a grant from the Bill & Melinda Gates Foundation enabled WHO/TDR to scale-up the programme over a four-year period and to engage the participation of additional pharmaceutical companies and product development partnerships (PDPs) as host institutions. An overview of fellows awarded throughout the programme period 1999–2013 is provided in Table 1.

**Table 1:** Overview of fellows awarded throughout the programme period 1999–2013. Altogether, 43 fellows were selected, however, for one fellow it was impossible to obtain visa. Hence, 42 were successfully placed, one withdrew due to conflict with supervisor at the host institution, and one was unable to return to home institution. Numbers of fellows for the period 1999–2013 sum up by the numbers indicated in bold, the years 2009–2012 are also shown as breakdown in light print. At the time of the evaluation, starting from August 2012, 27 fellows had completed the CDF programme.

Period of time	Number of fellows selected	Gender ratio (m/w)	Fellowship completed	Fellowship not completed	Reintegration successful	Reintegration not successful
**1999–2009**	**9**	**8/1**	**9**	**0**	**9**	**0**
2009–2010	7	6/1	7	0	6	0
2010–2011	4	3/1	4	0	4	0
2011–2012	7	6/1	6	1	7	0
**2009–2012**	**18**	**15/3**	**17**	**1**	**17**	**0**
**2012–2013**	**15**	**7/8**	**15**	**0**	**14**	**1**
**Summary:**						
**1999–2013**	**42**	**30/12**	**41**	**1**	**40**	**1**

The CDF programme provides qualified scientists from LMICs with three core supports: (i) a grant that covers the administrative costs of the fellowship, including attendance at one professional scientific meeting during fellowship; (ii) a 12-month assignment in the clinical department of a pharmaceutical company or PDP (including necessary training resources such as clinical supervision and mentoring); and (iii) an annual face-to-face alumni meeting and networking opportunities through a dedicated career development website [6]. The programme addresses the research fields of product development (PD) and clinical trials (CT), which are not widely taught at academic centres. Between 1999 and 2012, at the time when the evaluation was launched, 27 fellows had completed this unique programme, which facilitates the development of career elements essential to becoming a leader in clinical research. The programme’s ultimate goal is for fellows to return to their home institutions equipped with specialized skills, experience and access to vibrant networks within the international research community. Fellows benefit their home institutions and, by extension, their home countries, by pursuing high-level research careers and influencing their local environments.

As part of the TDR framework for performance assessment [7], two institutions–the Swiss Tropical and Public Health Institute (Swiss TPH, Basel, Switzerland) and the Barcelona Institute for Global Health (ISGlobal, Barcelona, Spain)–were commissioned to undertake an external and independent evaluation of the WHO/TDR CDF programme, “to evaluate the outcome and potential impact of the project in order to provide the evidence to assist on recommendations and future decision making.” This article summarizes the approach and results of the evaluation, and indicates how the programme could be adapted in the interest of further scaling-up and extension. Similar initiatives may benefit from the experience of the CDF programme.

## Methods

### Approach

In order to assess the WHO/TDR CDF programme in its various aspects, the evaluators employed a results-based monitoring and evaluation approach [8]. This encompassed an initial assessment of inputs, examined activities and outputs, and culminated in a review of outcomes and impact, for which indicators were defined. The evaluation was designed to assess the programme from the following four perspectives: i) Relevance: Does the CDF programme address relevant challenges, needs, and gaps for fellows and their home institutions and countries?; ii) Effectiveness: Does the programme deliver training and capacity development effectively?; iii) Efficiency: Does the programme implement its activities in an efficient manner?; and iv) Impact and Sustainability: Has the CDF programme contributed to developing clinical research and product development capacity for LMIC researchers, institutions, and countries, and will it continue to do so?

### Log frame

A log frame was developed encompassing the four aspects discussed above, including detailed descriptions and objectives for each of the dimensions to be evaluated, outputs and/or outcomes, and defining indicators and data sources (Table 2). This log frame underwent several rounds of external review and refinement before the development of evaluation tools used to collect data from programme participants.

**Table 2:** Main areas of the programme covered in the evaluation log frame.

**Strategy and management**
**Communication and marketing**
**Recruitment and selection process**
**Fellows’ learning experience**
**Relationship between host companies, fellows, home institutions, and CDF management team**
**Reintegration of fellows into their home institutions**
**Roles and responsibilities of the various stakeholders**
**Experiences and outcomes for the various stakeholders**

The initial evaluation design was hampered by the fact that benchmarks had not been established at the outset of the CDF programme, i.e. the “training needs” for home institutions’ purposes or “training gaps” of fellows. These baseline indicators could only be included in the evaluation as retrospective assessments by the survey participants. For this, the evaluation team developed prelisted ranges of possible bottlenecks, gaps, or professional activities for the persons filling in the survey to select from, with the opportunity to add others in free text. For instance, the evaluators surveyed fellows regarding their pre-fellowship skills and knowledge through a questionnaire.

### Sources

Open access TDR documentation and relevant websites as well as internal documents provided by CDF management served the evaluation team as written sources. The following groups of people involved in the programme were addressed in different ways: (i) the CDF management team at WHO/TDR, (ii) representatives of home institutions, (iii) representatives of host institutions, and (iv) fellows.

Using the log frame, three versions of a comprehensive questionnaire were developed to be administered to representatives of home institutions, host institutions, and fellows in order to retrieve information about various aspects of the programme from multiple perspectives (Table 3). Based on both the log frame and survey results, four versions of a focus group discussion catalogue were developed, one for each of the three surveyed groups and an additional one for CDF management.

**Table 3:** Elements addressed in the questionnaires.

**Admission process**
**Relevance to individual and institutional needs**
**Effectiveness in developing fellows’ research capacity**
**Fellows’ absence, return, reintegration, and impact**
**Roles and responsibilities**
**Programme management**
**Online resources**
**Long-term effect on collaboration and sustainability**

### Interviewees

An attempt was made to contact and invite all home institution, host institution, and fellow participants in the programme to participate in the evaluation (27 fellows, 16 host institution representatives, 25 home institution representatives, Table 4). Out of those successfully contacted, a fairly good response rate was achieved from programme fellows (about 78%). Host institution representatives were somewhat less responsive (56%). Responses from nine out of sixteen representatives gave perceptions ranging from critical to enthusiastic, as mirrored in the participation in the alumni meetings. In contrast, such diversity of views could not be obtained from representatives of home institutions where only three out of ten responded. The low response rate from home institution representatives (12%) was likely due to the lack of a specified focal person for the CDF programme or to the original programme liaison no longer being available. We must consider that results drawn from the home institution group may be biased in the sense that the ones complying were possibly the mentors most involved in the programme. However, this fact strongly left its mark in the recommendations for the programme’s future regarding the involvement of home institution representatives.

**Table 4:** Responsiveness of programme participants to the survey.

Participants	Number who responded to survey	Number successfully contacted for survey	Total number
**Fellows**	21	27	27
**Host companies’ representatives**	9	16	16
**Home institutions’ representatives**	3	10*	25

*In some cases contact person information was not available or was no longer correct, in other cases there was no focal person or coordinator to contact.

### Procedure

The evaluation was carried out between August 2012 and April 2013 and covered the programme from 1999 through 2012. In a first phase, documents were screened and the log frame developed, after which the CDF management team was interviewed to refine the evaluation strategy. Following a piloting phase, questionnaires were administered to the three participant groups. Analyses were followed by in-depth focus group discussions and interviews with representatives from all three groups and the CDF management team before the final report was drafted.

Questionnaires were administered online. The surveyed groups were adequately informed of the procedures, and general and personalized reminders were delivered in a timely manner. Quantitative responses were collected and compiled to inform the next phase of the evaluation, which included an in-depth analysis of survey results in order to determine which aspects of the programme would be qualitatively addressed through stakeholder interviews.

Preliminary and final results were discussed with all stakeholders involved (including the donor organization and CDF management) via telephone interviews and in-person meetings, and were presented during the third CDF programme alumni meeting. Results and recommendations from the evaluation were translated into a reporting document that was presented on various occasions from a ‘lessons learned’ standpoint. Results of the evaluation are also briefly described on the TDR website [9].

## Results

### Relevance

**Fig 1:**
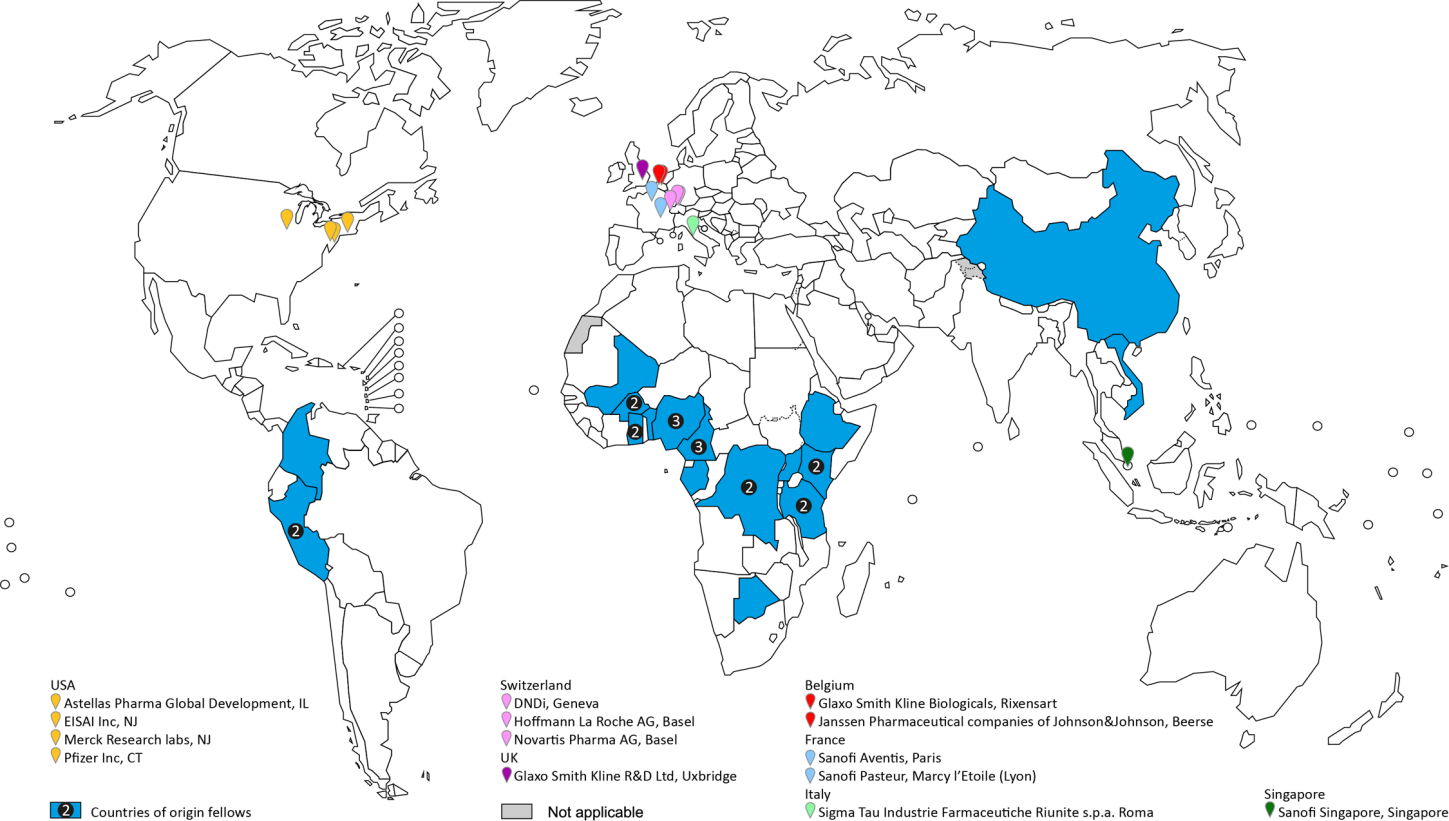
Distribution of fellows’ home countries and host institutions during the Career Development Fellowship (CDF) Programme between 1999 and 2012. Data Source: **World Health Organization**. Map Production: **WHO Graphics**. The boundaries and names shown and the designations used on this map do not imply the expression of any opinion whatsoever on the part of the World Health Organization concerning the legal status of any country, territory, city or area or of its authorities, or concerning the delimitation of its frontiers or boundaries. Dotted and dashed lines on maps represent approximate border lines for which there may not yet be full agreement.

The CDF programme exemplifies all elements included in TDR’s mission statement, mandate, and objectives. It is consistent with TDR’s strategic commitment to develop innovative knowledge, solutions, and implementation strategies on health needs [10–12], and translates these concepts into practice. The CDF scheme supports evidence-based decisions and so contributes to the development and implementation of new or improved interventions as well as to the translation of innovation, knowledge, solutions, and implementation strategies to policy and practice in addressing development goals and improving health in disease endemic countries. Applicants to the programme and the fellows selected fall into the scope of the programme based on their scientific backgrounds. Fellows originate from target countries and regions (Fig 1). Programme participants are placed at host institutions that contribute to the goals stated in the programme.

Fellows surveyed identified major bottlenecks for their home institutions and reported whether they were, in their view, addressed by the CDF programme (Table 5). Twelve persons (57%) estimated that the CDF programme addresses the bottlenecks of home institutions well or very well. The fellows’ placements were–through joint efforts–good matches between the needs of the fellows’ home institutions and the fellowship opportunity offered by the host company. In consequence of this perfect fit strategy, and due to a preference for high quality over quantity of participants, some CDF programme fellowship positions were not filled.

**Table 5:** Fellows (n = 21) identified major institutional bottlenecks and assessed the programme’s contribution. Bottlenecks were prelisted, with an option to add others in freetext.

Bottlenecks to the institution and country	Identified by # of fellows	Identified by % of fellows	Addressed by programme according to # of fellows	Addressed by programme according to % of fellows
**Funding**	**17**	**81**	**3**	**14**
**Facilities/Infrastructure**	**14**	**67**	**3**	**14**
**Staff knowledge/Capacity**	**14**	**67**	**15**	**71**
**Lack of collaboration with other institutions/companies**	**13**	**62**	**9**	**43**
**Lack of shared knowledge**	**11**	**52**	**7**	**33**
**Regulatory issues**	**10**	**48**	**7**	**33**
**Proposal development**	**9**	**43**	**10**	**48**
**Generating new ideas**	**8**	**38**	**5**	**24**
**Project management**	**8**	**38**	**8**	**38**
**Proposal writing**	**8**	**38**	**9**	**43**
**Administration**	**6**	**29**	**1**	**5**
**Documentation**	**6**	**29**	**3**	**14**
**Lack of analysis**	**4**	**19**	**1**	**5**
**Dissemination of results**	**3**	**14**	**0**	**0**
**Other cross-cutting skills**	**2**	**10**	**1**	**5**

Home institution representatives stated that the CDF programme led to alleviation of institutional scientific isolation, mainly through the networking aspect of the programme, which facilitates long-lasting relations through post-training communication and collaboration on several levels: between fellows, between fellows and their host institutions, and between fellows’ home institutions and the developing CDF programme network.

Bottlenecks identified by host companies encompassed institutional staff knowledge (8/9), project management ((7/9), and lack of collaboration with other institutions/companies (6/9). Host companies’ representatives reported–similar to the fellows’ perceptions–adequate targeting of the CDF programme to existing bottlenecks, both to theirs and the ones of the fellows. In addition, they valued the programme’s contribution to improving both “administration” and “documentation” higher.

Taken altogether, the evaluation found the CDF programme to be highly relevant with regard to TDR objectives and the programme’s own stated objectives.

### Effectiveness

The training components covered by the various fellowship placements include i) scientific knowledge (clinical pharmacology, good clinical practice, good laboratory practice, biostatistics, microbiology/molecular biology, medicine and clinical trial design), ii) technical skills (project planning and management, evidence-based study implementation, ethics and ethical clearance, regulatory compliance, monitoring and evaluation, quality control, meeting organisation and presentation), and iii) cross-cutting skills (see below).

CDF participants emphasized the following as major successes of the programme: i) a practical approach to addressing research skill needs on the scientific, technical, and cross-cutting levels; and ii) the involvement of the fellow in a wide range of professional activities through a strong link to the host company and to a larger international research network.

Mirroring the fellows’ individual research capacity needs, the pre-training gaps identified are summarized in Table 6. More than 90% of fellows reported that their skills and competencies in product development or clinical trials were either better (29%) or much better (62%) following their participation in the fellowship. The hands-on experience provided was highly valued. When asked whether they would prefer more theoretical training or more hands-on experience, 76% of the fellows chose the latter.

**Table 6:** Fellows (n = 21) identified individual pre-training gaps in skills and competencies and assessed the programme’s contribution. Gaps were prelisted, with an option to add others in freetext.

Personal pre-training gaps	Identified by fellows [number(%) of fellows]		Addressed by programme [number(%) of fellows]	
**Project management**	**17**	**81**	**20**	**95**
**Trial design**	**12**	**57**	**17**	**81**
**Clinical pharmacology**	**11**	**52**	**7**	**33**
**Good Clinical Practices / Good Lab Practices**	**10**	**48**	**18**	**86**
**Biostatistics**	**7**	**33**	**7**	**33**
**Microbiology or molecular biology**	**6**	**29**	**2**	**10**
**Ethics**	**4**	**19**	**17**	**81**
**Medicine**	**1**	**5**	**2**	**10**

In addition to scientific skills, fellows reported considerable improvement in cross-cutting skills such as (in %): study implementation (90), regulatory issues (90), documentation (86), monitoring & evaluation (81), project planning (81), management and leadership (76), problem-solving (71), quality control (67), ability to acquire new knowledge (62), collaborative practice (62), administration (57), social networking (48), and evidence-based implementation (38).

The networking aspect of the CDF programme was brought forward through three platforms, i) the online platform and ii) the alumni network, and iii) alumni meetings.

Initially developed as a communication platform site for fellows and launched under the name “Continuing Professional Development”[13], the online portal became crucial for programme participants. It soon evolved into a career development tool now known as the Professional Membership Scheme (PMS), which all fellows are invited to join. It was built in partnership with WHO-TDR in order to capture the development of core competencies as current and former fellows progress through their careers. The PMS is now embedded in the Global Health Network [6], a virtual professional community comprising a collection of interconnected specialist research sites linked through a digital hub, much like an online science park. Its success was one of the positive unintended outcomes from the CDF programme noted by the evaluation team. During the period from mid-October 2012 to mid-June 2013, PMS web pages received an average of 25 unique visits and 248 views per month from various stakeholders. Fellows reported that some of the most important uses of the website are networking (64%), retrieving programme information (64%), and searching for advanced training options (36%). Interestingly, 58% of fellows reported that the alumni network helps them to alleviate scientific isolation. Growing attendance at the annual CDF alumni meeting, which brings together CDF management, fellows and both host and home institution representatives, may be seen as an indicator of increased networking activity (Table 7).

**Table 7:** Attendance at CDF alumni meetings.

Attendance at CDF Alumni Meetings		
Year	*% of former fellows*	% of host companies*
**2010**	**90,5 (19/21)**	**50 (9/18)**
**2012**	**96,3 (26/27)**	**69 (11/16)**

*host companies were not reimbursed to attend the alumni meetings from the programme funds

Taken together, the results show evidence that the CDF programme delivers the intended training and capacity development in an effective manner.

### Efficiency

A clear measure of the efficiency of the CDF programme was the successful transition from Phase I to Phase II and the expansion of the programme to include more fellows, host companies, and home institutions from LMICs. Activities were implemented efficiently. In general, programme deadlines were met, although delays caused by external factors like visa application procedures and contract processes in companies, were seen in all recruiting rounds. The selection process was identified as very transparent by all stakeholders involved. Each round of selection has seen more eligible applications (minimum double) than positions offered. CDF management has opted for quality in each round, leaving positions empty rather than accepting fellows that are not optimal matches. The support and flexibility of host institutions to facilitate the integration of the fellows, both at the cultural and working levels, is noteworthy.

Once training placements are over, a smooth reintegration of fellows is necessary in order for LMIC-based home institutions to reap the benefits of the CDF programme. However, 38% of fellows reported that their reintegration was “problematic,” and three fellows reported that problems with re-entry were not resolved, despite the initial agreement between home institutions and TDR to reintegrate fellows at least at their previous level of employment. Such findings emphasize the home institution stakeholders’ crucial role as participants and supporters of the fellowship programme in order for it to sustain long-term impact.

### Impact and sustainability

This evaluation reviewed the short-and mid-term impacts of the CDF programme. There were three major indicators for impact: (i) enhancement of research/scientific activities; (ii) improvement in research environment at institutional and national level; and (iii) engagement in high-level scientific collaboration. The indicators include the amount and quality of fellows’ training, research, and networking activities; number of publications and conferences; and involvement in PD and CT post-training.

A crucial assessment of the impact and sustainability was the involvement in high-level scientific activities towards the end of and after the CDF fellowship period. The data in Table 8 suggest that fellows are equipped with skills for leadership and an ability to conduct projects in an international context. An interesting aspect to highlight is the level of involvement of fellows in national and international collaborations both during (52%) and after (81%) the training period.

**Table 8:** Participation of Fellows (n = 21) in professional activities. Since we lack pre-training data these results cannot be viewed as evidence of professional advancement attained through the CDF programme. Professional activities were prelisted, with an option to add others in freetext.

Professional activity	number of fellows	% of fellows
**Participation in PD/CT Projects**		
**Submission of at least one protocol for ethical approval**	**14**	**67**
**Participation in at least one clinical trial**	**13**	**62**
**Participation in at least one PD project**	**10**	**48**
**Participation in a leading role in at least one PD/CT project**	**9**	**43**
**Participation in Grant Applications**		
**Participation in at least one international grant application**	**12**	**57**
**Participation in at least one national grant application**	**3**	**14**
**Receiving a Grant**		
**Received an international grant**	**7**	**33**
**Received a national grant**	**2**	**10**
**Dissemination of Results**		
**Established additional national or international research collaborations after completion of training period**	**17**	**81**
**Submitted at least one manuscript for publication**	**15**	**72**
**Participated in a national or international meeting or conference related to PD/CT**	**14**	**67**
**Established additional national or international research collaborations**	**12**	**57**
**Had at least one publication**	**9**	**43**

Anecdotal (at this stage) cases showed that sustainability was supported best when home institutions gave a high level of attention to the post-training reintegration period of their fellow. Also, first evidence indicates an important role of the home institution key persons towards harmonized communication between fellows, host, and home institution personnel.

So far, the CDF programme has contributed to developing PD and CT capacity for LMIC researchers, institutions, and countries, and has made good progress towards strengthening research capacity.

### Recommendations

The evaluation highlighted the following general recommendations: i) continue and expand the CDF programme, ideally in partnership with other research organisations with similar supporting objectives; ii) develop a reintegration process that includes a re-entry grant scheme, overlapping fellows at host companies, and securing academic credit for programme participation; and iii) involve home institutions, primarily by better understanding and defining the roles and responsibilities of the different stakeholders in the CDF programme.

## Discussion

One fundamental cause of LMIC’s limitation in terms of clinical research remains the insufficient number of experienced researchers with: i) necessary knowledge and skills, ii) access to scientific networks, and iii) the confidence to design and lead their own research programmes [14]. The present external evaluation found that the CDF programme, as designed, enables fellows to acquire essential knowledge and skills. Many fellows reported that their skills and competencies for PD and CT are (much) better following the fellowship, and that the hands-on training they received was critical for their career development.

However, other institutional-level factors such as: i) research infrastructure, ii) the translation of individual capacity into institutional research capacity strengthening, and iii) an environment that allows trained researchers to stay in or return to low and middle income home countries, remain key [2, 3, 15]. The CDF programme stipulates the return of fellows to their home institution for at least one year after completion of the fellowship period. All but one (at the time of the submission of the manuscript, 41 fellows had completed their fellowship, Table 1) were able to comply with this stipulation. Similar findings have been made for the majority of other TDR postgraduate grantees [16]. The evaluation found that the programme has created a context that enables former fellows to build on their network of peers, mentors, and supervisors. As such, the CDF programme is surely not contributing to ‘brain drain’.

Through the evaluation, it became clear that the home institution has a central role in ensuring successful reintegration. It is the responsibility of home institution representatives to provide an appropriate environment in which returning fellows can share their new skills. The rather low percentage of fellows who reported improvement in evidence-based implementation is of concern, emphasizing the crucial role of the home institution. In cases where home institutions valued the return of their fellow as an opportunity to develop their institutional, local, and national research environment, they were able to successfully translate the individual fellowship into sustainable RCS. It is, however, the fellows’ responsibility to commit to sharing what they have acquired through the fellowship: experience, expertise, methods and tools, teaching skills, contacts, and networks.

The fellowship experience does not end upon return to a home institution. On the one hand, regular alumni meetings allow CDF programme peers and mentors to meet. On the other hand, the web-based portal is continuously and increasingly being utilized, particularly following its evolution into the PMS (embedded in the Global Health Network and used by many professionals in health-related research and development), through which relevant career competencies can be documented and displayed. Rooted in, but grown beyond application within the CDF programme only, the PMS was revealed by the external evaluation as an unintended outcome that heavily contributes support to CDF alumni and, beyond, to the international clinical research community. Above all, both the personal and work-related contacts between fellows and host institutions made through the programme prove to be major sources for continued and future collaborations (for 57% of fellows) across disciplines and sectors, nationally and internationally. Surely, capacity-building approaches are most promising when driven by the spirit of shared responsibility and guiding principles shared by donors and partners from different sectors in the North and South [5, 17–20].

The period (post-2013) following the independent evaluation of the WHO/TDR CDF programme, has seen considerable progress. With regard to the successful reintegration of fellows, a three-step strategy has been developed including: (i) define clear responsibilities, and a mentor, at the home institution; (ii) campaign at home institutions to stress the relevance of returning fellows and how they add value to the institutional, national, and regional research strategy; and (iii) design a reintegration scheme process to support fellows’ return to their home institutions. During the 1999–2012 period covered by the evaluation, 27 fellows from 16 different countries, mainly from Africa with a minority from Asia and Latin America, participated in the programme. At the time of submission of this manuscript, an additional 16 fellows have been trained in 10 host institutions, including three PDPs and two research institutions (one of them located in Zimbabwe). The programme has expanded to a total of 42 current selected fellows, 18 host organisations, and now covers eight different disease areas. More qualified female candidates have been encouraged in the call description and during the application period to enter the selection process in order to adjust the gender balance in the long term.

In the course of the evaluation, selected fellows raised both during interviews and alumni meetings that any form of internationally recognized academic accreditation of the CDF would add to career prospects. Also, on sub-Saharan African institution is not sending candidates due to lack of academic recognition and instead send their staff for academic degrees. The issue of accreditation remains unresolved for the moment as the programme will need to make efforts to meet the lifelong learning requirements set out in the Bologna Declaration for acquisition of credits in non-higher education contexts [21].

Subsequent to the evaluation period, and in line with the evaluation’s recommendations, the new phase of the CDF programme was jointly launched in 2014 by TDR and the European & Developing Countries Clinical Trials Partnership (EDCTP), which signed a partnership agreement in March 2014 for a clinical development fellowship allowing both to be, “more efficient with the funding provided by our donors” [22]. Beginning this year, an additional 25 fellows per year will be placed at some 20 high-level product development organisations [23, 24] through this partnership. We look forward to the clinical research collaborations and networks that will emerge from this endeavour.

## Supporting Information

S1 Data External Evaluation Final Report. (PDF)
