# Supplementary material for: Research Capacity Strengthening in Low and Middle Income Countries – An Evaluation of the WHO/TDR Career Development Fellowship Programme
Source: PLoS Negl Trop Dis. 2016 May 25;10(5):e0004631. doi: 10.1371/journal.pntd.0004631 (PMC4880212; doi:10.1371/journal.pntd.0004631)
Supplement: S1 Data — (PDF) [file pntd.0004631.s001.pdf]

External Evaluation Final Report

Special Programme for Research and Training in Tropical Diseases (TDR)

Career Development Fellowship Programme

August 2013

Principal evaluators:

Dr Michael Käser
Swiss Tropical and Public Health Institute
Socinstrasse 57
CH - 4002 Basel, Switzerland
Tel: +41 612 84 8365
E-mail: m.kaeser@unibas.ch
www.swisstph.ch

Swiss TPH
Swiss Tropical and Public Health Institute

Dr Núria Casamitjana
Barcelona Institute for Global Health
Rosselló 132
E - 08036 Barcelona, Spain
Tel: +34 932 27 1806
E-mail: nuria.casamitjana@isglobal.org
www.isglobal.org

ISGlobal Barcelona Institute for Global Health

Contents

Section 1: Executive Summary ... 3
1.1 Highlighted Recommendations ... 3
1.2 Evaluation Observations ... 4
1.3 Additional Outcomes ... 6
Section 2: External Evaluation Overview ... 7
2.1 Background of the CDF programme ... 7
2.2 Objectives and Evaluators ... 7
2.3 Evaluation Methodology ... 8
2.4 Evaluation Phases ... 8
2.5 Evaluation Data Sources ... 8
2.6 Constraints of the Evaluation ... 10
2.7 Recommendations for Future Evaluations ... 10
Section 3: Results & Recommendations: Relevance ... 11
3.1 Consistency with the TDR Mission Statement and Mandate ... 11
3.2 TDR Strategy ... 11
3.3 DEC Development Goals ... 12
3.4 DEC Needs for PD/CT Research – "research bottlenecks" ... 12
Section 4: Results & Recommendations: Effectiveness ... 15
4.1 Expanding the CDF programme ... 15
4.2 CDF Programme Ability to Train PD/CT Professionals – addressing "training gaps" ... 17
4.3 CDF programme ability to increase DEC institutional capacity for PD/CT Research ... 20
4.4 Online resources and tools ... 23
4.5 Alumni networking and support ... 27
Section 5: Results & Recommendations: Efficiency ... 29
5.1 Budget management ... 29
5.2 Calls, applications, selection, and milestones ... 30
5.3 Addressing issues and challenges ... 31
5.4 Oversight and management of the CDF programme ... 32
5.5 Partner's roles and responsibilities ... 33
5.6 Interrelations ... 34
5.7 Reporting ... 36
Section 6: Results & Recommendations: Impact and Sustainability ... 37
6.1 Impact ... 37
6.2 Programme Sustainability ... 38
6.3 Gender and Geographical Distribution ... 40
Section 7: Unintended Outcomes ... 42
Annexes ... 43

Abbreviations

BMGF	Bill and Melinda Gates Foundation
CDF	Career Development Fellowship
CPD	Continuing Professional Development
DEC	Disease endemic country
EDCTP	European & Developing Countries Clinical Trials Partnership
GHN	Global Health Network
GCP/GLP	Good clinical practice / good laboratory practice
LMIC	Low and middle income countries
PD/CT	Product development / clinical trial
PDP	Product development partnership
PMS	Professional Membership Scheme
RCS/KM	Research capability strengthening and knowledge management team
STAC	Scientific and Technical Advisory Committee
TDR	Special Programme for Research and Training in Tropical Diseases
WHO	World Health Organisation

Annexes

A. List of participating fellows, home countries, host companies, and research topics

B. Screen shot: TDR Website

Section 1: Executive Summary

The Career Development Fellowship (CDF) programme on clinical research and development is conducted by the World Health Organization (WHO)'s Special Programme for Research and Training in Tropical Diseases (TDR), in collaboration with pharmaceutical companies and is supported by the Bill and Melinda Gates Foundation (BMGF). CDF postgraduate fellows from hospitals, academic and research institutions (home institutions) in disease endemic countries (DECs) of low and middle income are selected to spend 12 months working in the clinical department of a host pharmaceutical company or product development partnership (host company). The CDF programme is intended to develop the individual capacity and careers of researchers, which is in turn expected to have impact on their home institutions and countries.

Following its inauguration in 1999, the CDF programme developed from an initial Phase I to a more ambitious Phase II. To date, 27 fellows from 25 different home institutions have participated in the programme – nine in Phase I and 18 in Phase II. Altogether, 16 host companies have offered training positions.

The results-based external evaluation assesses the programme's overall performance, identifies strengths and weaknesses, and highlights areas for improvement and future development. The evaluation was organised into the four categories *Relevance*, *Effectiveness*, *Efficiency*, and *Impact and Sustainability*. It was performed between August 2012 and April 2013 by a team of evaluators from the Swiss Tropical and Public Health Institute (Swiss TPH), Basel, Switzerland, and the Barcelona Institute for Global Health (ISGlobal), Barcelona, Spain.

The evaluators noted the following limiting factors:

1. Accessibility and responsiveness of programme participants to survey and interview requests varied from very good (fellows) to intermediate (host companies). Home institutions showed the lowest response rate.

2. Baseline and benchmark indicators: Creating mechanisms for on-going data collection will be useful to prepare for the next evaluation process.

3. Documentation: Systematic CDF data documentation would allow a more precise analysis of the programme's development over time.

1.1 Highlighted Recommendations

The evaluation provided evidence that the CDF programme has an impact on a broad range of factors relevant to achieving the programme's goals and objectives, with ample future potential for continued growth and extension. The CDF programme is well-placed to continue growing and developing successfully. The following general recommendations summarise more detailed suggestions made in the main report and are intended to support continued growth.

1. **Continue and expand the CDF programme.**
   This evaluation supports both continuing the CDF programme and expanding it in terms of the number of participating fellows, home institutions and host companies.

2. **Develop a reintegration process.**
   Long-term support to the fellows can be strengthened by focusing attention on the following key points: a re-entry grant scheme, overlapping fellows at host companies, securing academic credit for the programme and encouraging more active and widespread use of the TDR Fellows website and the Professional Membership Scheme (PMS), all initiatives that could provide support to fellows as they reintegrate into their home institutions and continue on their path of professional development.

3. **Involve home institutions.**
   More active involvement on the part of home institutions from the beginning of the programme, and better and more defined roles and responsibilities for home institutions could greatly improve their commitment to the programme. It would also enhance the benefits that home institutions enjoy through their participation in the CDF programme and the translation of individual capacity into institutional capacity once their fellows return.

1.2 Evaluation Observations

Relevance

In addition to generally supporting the Millennium Development Goals, the CDF programme is highly relevant to both the TDR mission statement and to TDR's mandate and strategy for research capacity strengthening. The programme responds well to TDR's commitments to develop innovative knowledge, solutions, and implementation strategies for improvement of health in disease endemic countries. The evaluation found that the CDF training programme does a good job of addressing research bottlenecks for home institutions and meeting training gaps for fellows.

Effectiveness

The CDF programme made a successful transition from Phase I to Phase II, expanding to include more fellows, hosts companies and home institutions. Participants continued to support the programme goals and objectives and were willing to help guide the CDF programme towards an improved implementation.

Despite goals for expansion of the programme, the CDF maintained an emphasis on quality during Phase II. The fellow selection process is generally well-defined and transparent and, although two candidates were unable to take up their positions, those that have been able to join host companies have successfully completed the programme. Fellows reported that the CDF training programme responded well to their needs –in terms of both professional competency development and improving cross-cutting skills. Fellows also liked the emphasis on hands-on training.

Reintegration into the home institution is sometimes a difficult experience for CDF fellows. Although all fellows who responded to our survey were able to take leave from institution for the entire duration of the programme, and of course there are cases where returning fellows have had an impact on their home institution environment, it is not clear that home institutions are always making full use of their CDF fellows' new skills upon their return.

An alumni support and networking programme (mostly via telephone calls and e-mails, but also via the dedicated website) is in place, although improvements could be made to strengthen the network. Two in-person alumni meetings have been held and more meetings would support connections and collaborations amongst programme participants. The evaluation results make it evident that the inclusion of home institution representatives in alumni meetings could be beneficial for the programme. The programme website, which could serve as a hub for communications, is currently underused by host companies and home institutions.

A forthcoming marketing and communications strategy planned by TDR could bring more users to the online platform, as well as energise the programme expansion and recruitment process. Such a strategy would include outreach to host companies as well as home institutions and fellows. It would involve a survey of other similar programmes and a profile of possible participants, as well as a marketing action plan for promotion of the CDF programme.

We recommend a broader marketing strategy for the excellent Professional Membership Scheme (PMS) online tool, a resource that has its roots in, but has grown beyond, the CDF programme. It is a high-quality platform that should be used by more professionals in this field and offers a great support opportunity for fellows and others during and after the training programme. Specifically, the evaluation indicates the need to create incentives for increased website usage, harmonisation of the two web platforms and regular communication with CDF programme participants, informing them about the characteristics and benefits of the online tools.

Efficiency

We suggest making improvements to reporting and documentation. On a variety of fronts, principally annual budget, oversight and management commmittes, alumni meetings, and fellows' progress reports, the CDF programme could benefit from more thorough documentation.

The overall programme budget, divided into three tranches plus one extension, and including relevant overheads and management costs, allowed for training of 35-40 CDF fellows by the end of 2014 (this evaluation does not cover the last recruiting round and therefore only documents 27 fellows). Per fellow costs vary greatly (based on country of origin and of placement) and the BMGF has been very flexible on this point.

In general, programme deadlines have been met despite the organisational challenges inherent to the programme. Some delays were expected in recruiting (due to legal procedures) and placement (mainly due to visa issues). In each round there have been more applicants than positions offered, which has allowed for shortlisting. CDF management has opted for quality rather than quantity and in some cases has left positions empty rather than select a fellow who is not an optimal match for the opportunity offered by a host company.

Communication between CDF management, host company mentors and fellows is generally efficient. Fellows reported that CDF management was easily accessible when approached and that all administrative issues were addressed, although some problems having to do with visas and contracts were difficult to resolve.

Impact and Sustainability

The impact of the CDF programme at an individual level reaches a level of high satisfaction. Scientific isolation is also addressed through the fellowship, through connections at the host institution and alumni activities. Although we lack information on the translation of impact at the institutional and country level, there is anecdotal evidence that returned fellows make a difference for their home institutions.

In some cases there has been continued communication between fellows and host companies after the placement. Two-thirds of participating hosts would continue to offer positions for CDF fellows, one-third answered with "do not know", for various reasons. The commitment of hosts to the programme is essential for its long-term survival and growth.

The CDF programme will also need to consider options for the long-term funding of the programme, whether it continues to receive support from a single funder (BMGF) or seeks to diversify its sponsors. A proposed re-entry grant scheme for CDF programme graduates will also require additional funding. The

evaluation found that such a scheme could provide important support for fellows as they reintegrate into their home institutions.

1.3 Additional Outcomes

1. Focus on institutional continuity.

Strengthening the collaboration between CDF, host companies and home institutions will help to ensure partnerships that allow investment in long-term capacity building at the institutional and country level. It is also instrumental to furthering individual capacity building.

2. Development of an online resource.

The Continuing Professional Development (CPD) tool, which was created in the course of the CDF programme, was developed into the Professional Membership Scheme (PMS) that is currently used beyond the scope of this programme and in the wider context of the Global Health Trial website, a development which shows the pioneering character of the CDF programme.

3. Showcase for similar initiatives.

The European & Developing Countries Clinical Trials Partnership (EDCTP), inspired by CDF, has created a similar programme, evidence of CDF’s positive effect on another organisation. Both the evaluation team and the CDF management view this as an opportunity for collaboration, complementary activities, and mutual learning. The interest in academic credit expressed by some fellows opens up the possibility of a fruitful collaboration with an academic partner. We hope that the CDF management will explore this in the near future.

Section 2: External Evaluation Overview

The World Health Organisation (WHO)'s Special Programme for Research and Training in Tropical Diseases (TDR) organises the Career Development Fellowship (CDF) programme on clinical research and product development, in collaboration with pharmaceutical companies and with the support of the Bill and Melinda Gates Foundation (BMGF). As far as we are aware, the CDF programme has been the only initiative at the interface of health-related research in low and middle income countries (LMICs) and commodity-focused industry, although the model is now being taken up by other organisations (such as EDCTP). In the 14 years since its inauguration, the CDF programme has earned a positive reputation. Near the end of the second phase of the CDF programme, the TDR management requested an external evaluation to assess the programme's overall performance, identify strengths and weaknesses, and highlight areas for improvement and future development.

2.1 Background of the CDF programme

The CDF programme began in 1999 at TDR, in collaboration with GSK Biologicals Belgium. In 2008, with continued support from the BMGF, the CDF programme was scaled-up to increase the number of fellows and engage more pharmaceutical companies and product development partnerships as hosts. Since 2009, the CDF programme has been led by Dr Pascal Launois and a TDR team that focuses on individual capacity building.

CDF fellows are selected from hospitals, academic, and research institutions (home institutions) in disease endemic countries (DECs) to spend 12 months working in the clinical department of a host pharmaceutical company and/or product development partnership (host companies), developing their capacity to participate in and manage clinical research that meets international regulatory standards for product development for diagnostics, drugs, and vaccines against the infectious diseases that disproportionately impact LMICs. Upon completion of the fellowship, the fellow is expected to pursue a career and attain a leadership role in his/her field of expertise in his/her home institution and/or country of origin. In the mid to long term, the CDF programme is intended to develop the institutional capacity of fellows' home institutions so that they gain greater influence on the health research agenda and benefit from improved access to research resources for diseases affecting their populations.

To date, 27 fellows from 25 different home institutions have participated in the programme – nine in Phase I and 18 in Phase II. Sixteen host companies have offered training positions.

2.2 Objectives and Evaluators

The terms of reference identify the objective of this evaluation as, "to evaluate the outcome and potential impact of the project WHO/TDR/CDF in order to provide TDR and the donor (BMGF) the evidence to assist on recommendations and future decision making. TDR/ID: B20246". The evaluation covers: CDF programme strategy and management, communication and marketing, recruitment and selection process, fellows' learning experience, the relationship between host companies, fellows, home institutions and CDF management team, reintegration of fellows into their home institutions and experience, engagement and outcomes for all programme participants.

In order to ensure an unbiased assessment, the evaluation was carried out i) by institutions/evaluators external to the WHO and TDR, and ii) in concerted action between two independent institutions. The external evaluators selected were the Swiss Tropical and Public Health Institute (Swiss TPH), Basel, Switzerland, and the Barcelona Institute for Global Health (ISGlobal), Barcelona, Spain. The principal evaluators were Dr Michael Käser (Swiss TPH) and Dr Núria Casamitjana (ISGlobal). The evaluation was carried out between August 2012 and April 2013.

2.3 Evaluation Methodology

The evaluators employed a results-based monitoring and evaluation approach beginning with inputs, moving through activities and outputs, and culminating in outcomes, impacts and feedback. Indicators were defined for each output and/or outcome. Where possible, benchmarks were assigned. Data sources were identified for each indicator.

The evaluation was designed along four broad lines:

1. Relevance: Does the CDF programme address relevant challenges, needs, and gaps for fellows and their home institutions and countries?
2. Effectiveness: Does the programme deliver the intended training and capacity development effectively?
3. Efficiency: Does the programme implement the activities in an efficient manner?
4. Impact and Sustainability: Has the CDF programme contributed to developing clinical research and product development capacity for DEC researchers, institutions, and countries; and will it continue to do so?

2.4 Evaluation Phases

The evaluation was carried out in the following phases:

1. Design: Definition of outputs, outcomes, indicators, means of verification, data sources and data collection tools in consultation with TDR staff, selected fellows, host companies, and home institutions. Before moving to the next phase, the evaluation design was shared with TDR management and the BMGF and approved by TDR management.
2. Development of tools: Detailed development of questionnaires and interview outlines. Listing of individuals/institutions to be surveyed.
3. Data collection from interviews, documentation and other developed tools, and analysis: Analytical review, identification of factors that could improve the programme.
4. Reporting and Recommendations

Throughout the process the evaluators consulted with TDR management team.

2.5 Evaluation Data Sources

The data to support the evaluation process were drawn from the following sources:

Open access TDR documentation

1. TDR website (www.who.int/tdr)
2. *Strengthening research capacity where it's needed most*
3. *TDR at a glance – Fostering an effective global research effort on diseases of poverty* (WHO-TDR)
4. *TDR research for a changing world* (DVD)
5. *Making a difference*. TDR strategic plan 2012-2017.
6. *Innovation for health – research that makes a difference*. TDR biennal report 2010-2011
7. *BL2 Business Plan 2008-2013. Empowerment. Harnessing DEC excellence and leadership for effective application of health research* (May, 2007)
8. *Investing in health and development. Research capacity building in developing countries*. UNDP/World Bank/ WHO TDR 2003

Documents provided by the TDR management team

- « Proposal Scale-up of WHO Tropical Diseases Research (TDR) Clinical Career Development Fellowship programme » (29 August 2008)
- « TDR's Clinical Research Career Development Fellowship Programme » (v. 13 December 2011)
- « Terms of Reference to build an on-line continuing development scheme for clinical trialists working in global health »
- « Progress Report Scale-up of WHO Tropical Disease Research (TDR) Clinical Career Development Fellowship programme » (v. 12/2009)
- « Progress Report Scale-up of WHO Tropical Disease Research (TDR) Clinical Career Development Fellowship programme » (v. 1/2011)
- « Brief note on the meeting with Marie Paul Kieny to discuss CDF grant and LEG issues Thursday 01 December 2011»
- « Building Careers in Medical Research Through Partnerships and Knowledge Sharing »
- « Milestones » (Appendix Gates, 04/05/2005)
- « Milestones table and timeline modified » (24/02/2012)
- « Alumni Report »
- « Names » (list of programme participants)
- « List-CDF Gates » (list of programme participants)
- « CDF grantees » (list of programme participants)
- « Selection process »
- « Breakdown budget for 2013 »

Interviews with CDF programme management team

Repeated rounds of interviews, both in person and via phone calls and e-mail conversation, were carried out throughout the external evaluation.

Relevant websites:

- TDR Fellows website (www.TDRfellows.org)
- Global Health Trials website (www.globalhealthtrials.org/)
- Professional Membership Scheme website (https://globalhealthtrials.tghn.org/cpd/about/)

In-depth interviews with selected programme participants (fellows, host companies, home institutions, TDR management team, Global Health Network management team)

Surveys

Three similar surveys were designed and distributed to the three participant groups: fellows, host companies, and home institutions. Questions were divided into eight categories:

1. CDF programme admission process
2. Relevance of the CDF programme
3. Effectiveness of the CDF programme in developing fellows' research capacity
4. Fellows' absence, return, reintegration and impact
5. Roles and responsibilities
6. Programme management
7. Online resources
8. Long-term effect on collaboration and sustainability

Surveys were delivered by e-mail in an electronic format (Google-based) and were designed to take between 20-30 minutes to complete. Participants were informed that their responses would be reported without any personally identifying information.

2.6 Constraints of the Evaluation

Constraints for this evaluation included:

1. Lack of pre-defined benchmarks and indicators: Although the methodology applied here was results-led, this was qualified to the extent that benchmarks and indicators for the programme had largely not been specified in advance. Data collection up to the point of the evaluation has been minimal.

2. Accessibility and responsiveness of programme participants (fellows, host companies, home institutions) to interview requests and survey: Both the survey and interview requests received a very good response rate from programme fellows (about 78%). Host institutions (56%) were somewhat less responsive. In particular, we received a low response from home institutions. In part, this may be because there has been no clear focal person or point of contact for the CDF programme or "mentor / supervisor" for the CDF fellow established at many home institutions.

3. Possible bias from home institution survey results: Due to the very low response rate we must consider that data drawn from this particular survey may be biased. The opinion of the respondents to the survey may not be representative of the entire participants of the programme.

Participants	Number who responded to survey	Number successfully contacted for survey	Total number
Fellows	21	27	27
Host companies	9	16	16
Home institutions	3	10*	25

*In some cases contact person information was not available or was no longer correct. In the case of some home institutions there was no focal person or coordinator to contact.

2.7 Recommendations for Future Evaluations

1. Indicators and benchmarks: In order to ensure fruitful evaluations in the future, it will be important to develop indicators and benchmarks for all programme outcomes.

2. Data collection: It will be useful to begin the next evaluation process now by creating mechanisms for on-going data collection.

3. Documentation: More robust CDF programme documentation, and a systematic organisation of documentation and data, would allow a more precise analysis of the programme's development over time.

Section 3: Results & Recommendations: Relevance

"Relevance" addresses the CDF programme's consistency with the TDR mission statement, mandate and strategy. The evaluation was made by analysing TDR documents (including the mandate and mission statements, the strategic plan, other available TDR documents, the TDR website) and through interviews with the TDR and CDF management teams. This section also includes an evaluation of whether the programme's activities are relevant to product development / clinical trial (PD/CT) needs in DECs, evidence for which comes mostly from the perceptions of programme participants regarding important research needs and "bottlenecks" for DEC institutions.

3.1 Consistency with the TDR Mission Statement and Mandate

The CDF programme as designed is highly relevant to TDR's mandate and objectives. In fact, the programme addresses nearly all of the points raised by the mission statement and mandate and addressed in the TDR documents, "TDR research for a changing world" and "TDR at a glance", including:

- To foster an effective global research effort on infectious diseases of poverty that leads to health improvement
- To engage disease endemic regions and countries in setting the health research agenda and harmonizing the global response
- To strengthen the capacity of individuals and institutions in DECs to perform research related to their own priority health issues
- To promote the involvement of individuals, communities and societies in the use of research evidence to reduce the burden of endemic diseases in their countries
- To provide stewardship, knowledge and advocacy for research
- To empower poor countries to develop their own priorities
- To strategically increase country ownership in R&D
- To breech barriers to appropriate R&D, politically and nationally
- To strengthen countries' overall R&D capacity
- To be a valuable partner to many donor and implementing organisations
- To foster research efforts with DECs playing a pivotal role;
- To coordinate, support, and promote global efforts to combat infectious diseases of the poor and disadvantaged
- To catalyse improvements in global health key strengths
- To convene people, facilitate dialogue, and provide a neutral platform for scientific exchange and discussion
- To build sustainable networks of researchers and research institutions, with DEC researchers increasingly being leaders of activities
- To create partnerships on all levels including national governments, clinics in remote areas, private industries, disease control programs, academia, research institutions, and NGOs.

3.2 TDR Strategy

The programme also speaks to TDR's research capacity-strengthening mission and strategy, including:

- Research capacity strengthening through support of training, leadership development and project-related capacity building to strengthen the ability of countries and regions to respond to their own research needs.
- Gap analysis for agenda setting through promoting evidence-based priority setting to identify emerging needs in research and capacity strengthening.

- Partnership and engagement through collaborating with WHO, TDR co-sponsors and partners for harmonization and alignment with global health goals.

The CDF programme supports evidence-based decisions and so contributes to the development and implementation of new or improved interventions tested in DECs, in response to TDR's commitments to *develop* innovative knowledge, solutions, and implementation strategies that respond to health needs; and to *translate* innovation, knowledge, solutions, and implementation strategies to policy and practice in improving health in DECs.

3.3 DEC Development Goals

The CDF programme supports the attainment of development goals in so far as it contributes, at a broad level to:

- Achieving Millennium Development Goals;
- Supporting strategic priority setting, research planning and the research agenda;
- Supporting the development and improvement of drugs, diagnostics, and preventive strategies for NTDs, and identifying the best ways to use these tools;
- Bringing together leading experts; working closely with DEC researchers and institutions;
- Involving key partners to evaluate tools and strategies, in the lab, in the community and with local researchers;
- Empowering research leadership in developing countries;
- Encouraging research development in the context of health research programme and in partnership with high quality institutions;
- Building national capacity and commitment for involvement in clinical, operational, policy-related and social science research and control programmes;
- Tackling "the big three" (malaria, HIV/AIDS, TB) with innovative approaches;
- Working with DECs around the globe (Africa, Asia, South America); and
- Discovering and developing effective tools that are field tested in real-life settings.

3.4 DEC Needs for PD/CT Research – "research bottlenecks"

In the CDF programme proposal phase, emphasis was placed on the extent to which the CDF programme could meet the needs of DECs, with a focus on two different aspects: i) the key bottlenecks that the DEC-based home institutions face in their research and working environment, which are referred to as the "research bottlenecks"; and ii) the gaps in knowledge and skills of the fellow target group, which are here called the "training gaps". In the course of the initial evaluation design, however, the evaluation team realized that neither "research bottlenecks" nor "training gaps" had been defined or described. This made it difficult to benchmark the ability of the programme to address either the needs of the home institutions or the fellows. Therefore, an assessment of both the "research bottlenecks" and the "training gaps" was carried out during the course of the evaluation by asking both fellows, and home institutions and host companies mentors for their input via survey. The responses to these questions are presented in the following figures for "research bottlenecks" and are used as a baseline for assessing how the needs of home institutions were addressed by the programme. The responses to the questions addressing the "training gaps" will be presented in section 4.2 - CDF programme ability to train PD/CT professionals.

In the following tables, we summarize the findings on the institutional and country "research bottlenecks":

Major Bottlenecks from the Perspective of Fellows (n=21/27)		
Funding	17	81%
Facilities / infrastructure	14	67%
Staff knowledge / capacity	14	67%
Lack of collaboration with other institutions/companies	13	62%
Lack of shared knowledge	11	52%
Regulatory issues	10	48%
Proposal development	9	43%
Generating new ideas	8	38%
Project management	8	38%
Proposal writing	8	38%
Administration	6	29%
Documentation	6	29%
Lack of analysis	4	19%
Dissemination of results	3	14%
Other cross-cutting skills (e.g., social networking, management and leadership)	2	10%

Fellows reported that, in their view, most bottlenecks were addressed, to a degree, by the CDF programme training.

Many fellows find that these bottlenecks are addressed by the CDF programme (Fellows' survey n=21)	Few fellows find that these bottlenecks are addressed by the programme (Fellows' survey n=21)
• Institutional staff knowledge (15/21)<br>• proposal development (10/21)<br>• proposal writing (9/21)<br>• lack of collaboration with other institutions/companies (9/21)	• administration (1/21)<br>• documentation (3/21)<br>• facilities/infrastructure (3/21)<br>• funding (3/21)

Fellows who responded to the survey were predominantly positive about the overall ability of the CDF training programme to address the needs of their home institutions.

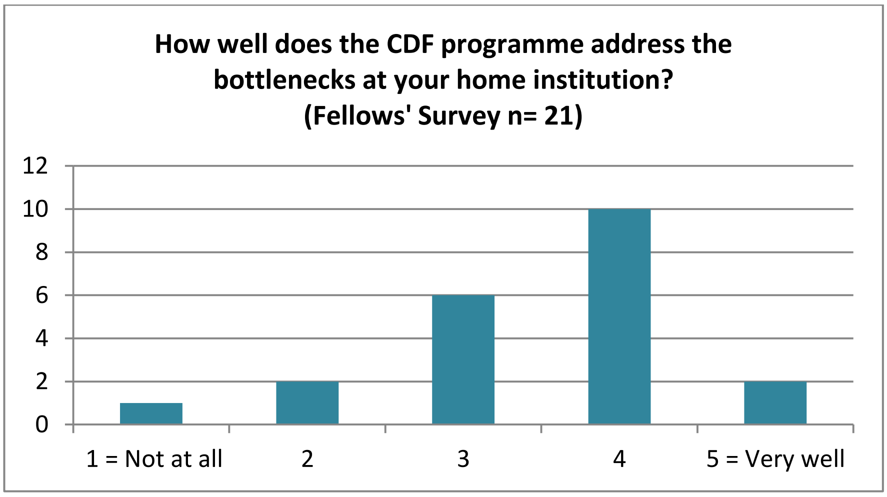

Host companies reported similar perceptions to fellows, although they valued the programme's contribution to improving "administration" and "documentation" higher.

Many host companies find that these bottlenecks are addressed by the CDF programme (Host survey n=9)	Few host companies find that these bottlenecks are addressed by the programme (Host survey n=9)
• institutional staff knowledge/capacity (8/9)<br>• project management (7/9)<br>• lack of collaboration with other institutions/companies (6/9)	• facilities/infrastructure (1/9)<br>• funding (1/9)<br>• lack of analysis (1/9)<br>• generating new ideas (2/9)<br>• proposal writing (2/9)

The three home institutions that responded to the survey reported a positive assessment of the match between their needs and the CDF programme as well.

RECOMMENDATION: **Assess the specific research bottlenecks** for home institutions and DECs **and training gaps** for individual fellow target groups at the stage of selection and entry into the programme to be used as a baseline for evaluation activities.

Section 4: Results & Recommendations: Effectiveness

Effectiveness was approached from diverse angles including programme development, training capacity and alumni, and web-based services.

4.1 Expanding the CDF programme

Phase I of the CDF programme (1999-2008) was carried out by TDR in cooperation with one pharmaceutical company. One measure of the effectiveness of the CDF programme was the successful transition from Phase I to Phase II and the expansion of the programme to include more fellows, host companies, and DEC-based home institutions.

Overview

The CDF programme developed new links with DEC-based home institutions, resulting in more opportunities for future fellows. At the same time, the programme successfully broadened the number of participating host companies so that more fellows could be placed during Phase II. Although the intention was to increase the size of the programme in Phase II, the focus continued to be on maintaining the quality of fellows.

Number of participating fellows, host companies and home institutions

The number of participants increased from Phase I to Phase II.

Number of CDF Programme Participants – Phase I and Phase II

	Phase I (1999 – 2008)	*Phase II (2009-2012)*
**Fellows**	9	18
**Host companies**	1	16
**Home institutions**	9	18

Five host companies and two home institutions participated multiple times in the CDF programme, demonstrating a commitment to the programme. This was true even though some of the host companies who chose to repeat participation had disappointing first experiences with fellows who did not fulfill expectations. This may indicate that hosts viewed the programme as being in a trial-and-error phase of development but continued to trust in the programme goals and objectives. They were willing to participate in this phase and to help guide the CDF programme to a more successful implementation.

Application of qualified candidates and selection process

In Phase I, the number of positions offered by the host company involved corresponded to the number of fellows finally placed. However, in Phase II, the number of positions offered by the new host companies exceeded the number of fellows finally placed by two companies: one fellow had visa problems and was not able to attend the programme and the other fellow withdrew from participation for unknown reasons.

As it is shown in the following table, the number of applications exceeded the number of open positions in the calls issued in 2009, 2010, and 2011.

CDF Programme Applications vs. Positions Offered

	Number of eligible applications	*Number of positions offered*	*Number of fellows selected*
**2009**	18	8	6
**2010**	19	7	5
**2011**	33	7	7

During the selection process, candidates were shortlisted based on their qualifications. In general, these shortlists held twice as many candidates as there were available positions. To date, all fellows who have been placed with host companies have successfully completed the CDF programme training.

The current call (not included in this evaluation) was issued in fall 2012. There were 125 candidates for 17 positions. Thirteen of the positions were filled. There has been a larger pool of sufficiently qualified candidates applying to the programme, allowing for an increasingly selective admission process and better qualified fellows, and, ultimately, leading to more and better qualified fellows entering the CDF programme.

The CDF programme selection process has been clearly defined and selection criteria are used to ensure the placement of qualified candidates (see Section 5.2). This process is driven by the CDF management team in a completely transparent way. CDF management provides a list of candidates to participating host companies. The host company can then contact and interview candidates from this list (via telephone). Once accepted, the candidate decides whether he or she will accept the placement.

A marketing and communications strategy of the CDF programme is under development. This has the potential to greatly improve the distribution of information about the programme, support development of new CDF programme partners and assist in candidate recruitment. Thus far, most surveyed host companies learned about the programme via a direct approach by TDR management. Fellows who responded to the survey reported that they found the call online or through a colleague.

The CDF programme has became increasingly well known and information about it is now distributed through a variety of e-mail lists and websites with information boards or links.

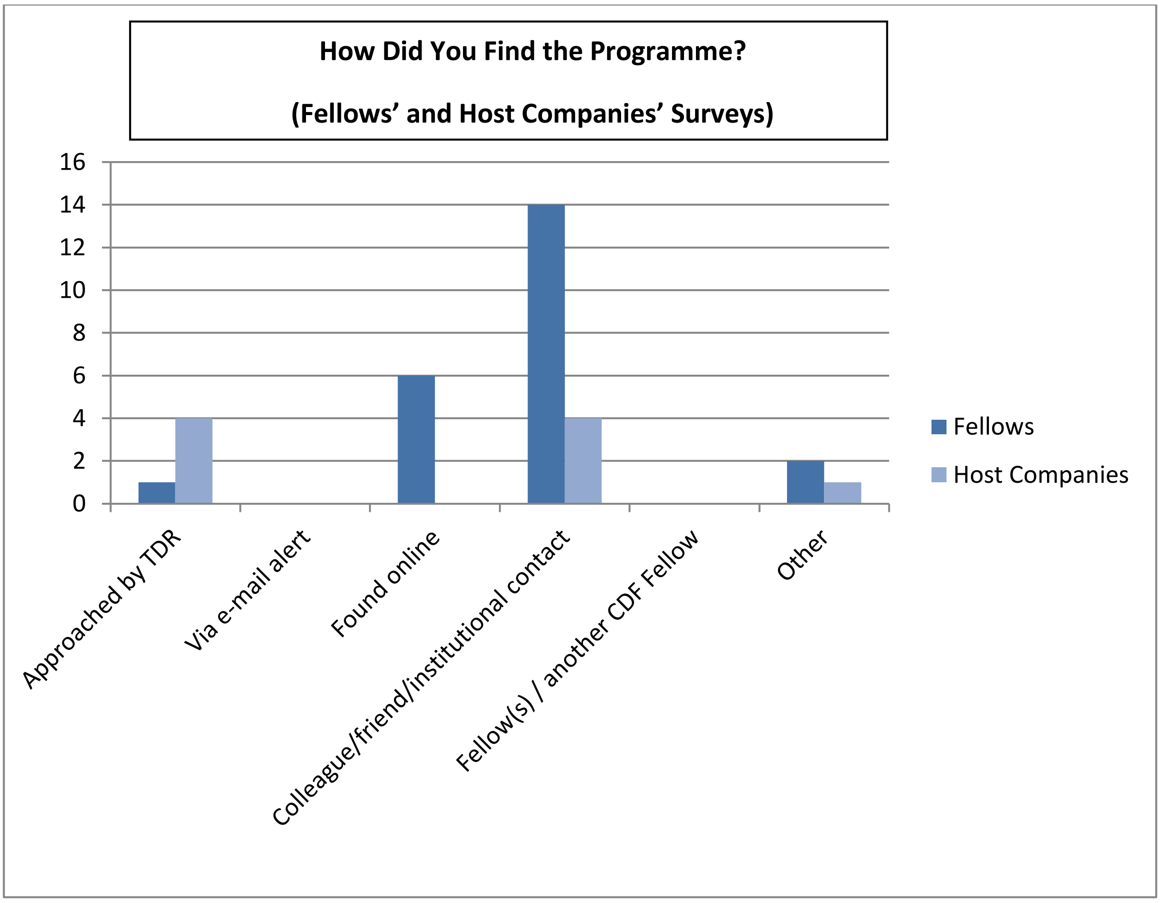

RECOMMENDATION: **Involvement of host companies**

Continue to involve host companies in the selection process and in the determination of the fellow's scientific topic. Continue ongoing and regular communication with host companies and invite them to participate in alumni meetings. Provide host company mentors with supporting information on the programme's goals, objectives and activities, giving them material to share with their company's management in order to justify the investment of resources and time.

RECOMMENDATION: **Marketing and communications strategy**.*

Increased distribution of the call has led to an increased applicant pool. The proposed **marketing and communications strategy** should be created and implemented. This strategy could include an analysis of the potential market of host companies as well as home institutions. It could also involve the **research into information platforms, fora, networks and online distribution lists**, etc. that are used by potential CDF fellow candidates. AFANET, ASPHA and tropEd are potential partners, among others.

Alumni fellows reported that they found the CDF programme through the following channels:

- From a colleague/friend/institutional contact: 14 (67%)
- Online: 6 (29%)
- A minority of fellows were either approached by TDR or found the call through other routes.

**In the fourth round of recruiting (not included in this evaluation) an extended portfolio of call information distribution was implemented. The CDF management received 125 applications from eligible candidates for 17 available placements, which provides an excellent ratio for appropriate shortlisting.*

4.2 CDF Programme Ability to Train PD/CT Professionals – addressing "training gaps"

The CDF programme was evaluated for its ability to provide training to increase the individual capacity of fellows and that responds to the needs of the DEC-based home institutions. In the following, we are referring the analysed gaps in knowledge and skills by the fellow target group, which is here called the "training gaps" (see definition in section 3.4).

Increased DEC Individual Capacity

More than 90% of fellows reported that their skills and competencies for PD/CT were either "better" (29%) or "much better" (62%) following their participation in the CDF programme. Altogether, 95% of survey respondents think that their skills and competencies in GCP/GLP were "better" or "much better" at the end of the programme. Fellows reported that they had gained skills in a range of fields.

The comparison below provides evidence that the gaps described by the fellows were addressed appropriately through the training in the most cases. And, in some cases, additional training was provided even where the fellows had background preparation (such as in "trial design", "project management" and "GCP/GLP (good clinical practice / good laboratory practice)". We see an unmet need for "clinical pharmacology" training. Eleven (52%) fellows reported they lacked training and only 7 (33%) reported

receiving it. For "ethics," 17 (81%) reported receiving training and only four reported a lack of prior experience. Ethics was not identified as a pre-CDF training gap.

Personal pre-"Training Gaps" in Skills and Competencies and Training Received Through the CDF Programme (Fellows' Survey; n=21/27)

	Lacked training prior to CDF		Received training at CDF	
**Project management**	17	**81%**	20	**95%**
**Trial design**	12	**57%**	17	**81%**
**Clinical pharmacology**	11	**52%**	7	**33%**
**Good Clinical Practices / Good Lab Practices**	10	**48%**	18	**86%**
**Biostatistics**	7	**33%**	7	**33%**
**Microbiology or molecular biology**	6	**29%**	2	**10%**
**Ethics**	4	**19%**	17	**81%**
**Medicine**	1	**5%**	2	**10%**
**Other**	1	**5%**	1	**5%**
**None of the above**	0	**0%**	0	**0%**

The hands-on experience provided by CDF training was valued. Asked whether they would prefer more theoretical training or hands-on experience, 76% of surveyed fellows chose hands-on experience. This result supports CDF management's decision to eliminate one month of theoretical training at TDR that was included in the programme during Phase I.

Half of fellows who responded to the survey would have liked to extend the CDF programme training period beyond 12 months.

Development of cross-cutting skills for PD/CT project

In addition to improved scientific skills, fellows reported improved cross-cutting skills to a considerable extent. These are essential skills that complement PD/CT and laboratory work for the development and implementation of projects.

Cross-cutting Skills Gained Through CDF Training (Fellows' Survey; n=21/27)

**Study implementation**	19	**90%**
**Regulatory issues**	19	**90%**
**Documentation**	18	**86%**
**Monitoring and evaluation**	17	**81%**
**Project planning**	17	**81%**
**Management and leadership**	16	**76%**
**Problem-solving**	15	**71%**
**Quality control**	14	**67%**
**Ability to acquire new knowledge**	13	**62%**
**Collaborative practice**	13	**62%**
**Administration**	12	**57%**
**Social networking**	10	**48%**
**Evidence-based implementation**	8	**38%**
**Other**	0	**0%**
**None of the above**	0	**0%**

"Production" of trained professionals

In the years one through three, following their return to their home institution, the participation of fellows in PD/CT activities varied:

Participation of CDF fellows in professional activities (following completion of programme, unless otherwise noted) (Fellows' survey; n=21/27)

Participation in PD/CT Projects	Number of fellows who responded to the survey who have participated in activity	Percentage of fellows who responded to the survey who have participated in activity
**Submission of at least one protocol for ethical approval**	14	**66,5%**
**Participation in at least one clinical trial**	13	**62%**
**Participation in at least one PD project**	10	**47,5%**
**Participation in a leading role in at least one PD/CT project**	9	**43%**
**Participation in Grant Applications**		
**Participation in at least one international grant application**	12	**57%**
**Participation in at least one national grant application**	3	**14%**
**Receiving a Grant**		
**Received an international grant**	7	**33%**
**Received a national grant**	2	**9,5%**
**Dissemination of Results**		
**Established additional national or international research collaborations after completion of training period**	17	**81%**
**Submitted at least one manuscript for publication**	15	**71,5%**
**Participated in a national o international meeting or conference related to PD/CT**	14	**66,5%**
**Established additional national or international research collaborations during the training period**	12	**57%**
**Had at least one publication**	9	**43%**

Since we lack pre-training data we cannot view these results as evidence of professional advancement attained through the CDF programme. Still, the data suggest that CDF-trained professionals are equipped with skills for leadership and an ability to conduct projects in the international context. An interesting aspect to highlight is the high level of involvement of fellows in national and international collaborations both during (52%) and after (81%) the training period.

RECOMMENDATION: In order to support a training programme that responds the needs of PD/CT professionals, the CDF Programme should **seek academic accreditation**.

RECOMMENDATION: **Assess the specific research bottlenecks** for home institutions and DECs **and training gaps** for individual fellow target groups at the stage of selection and entry into the programme to be used as a baseline for evaluation activities. Also, share this information with host companies to improve the learning objectives developed for the training period.

RECOMMENDATION: **Use the selection processes to involve home institutions in assessing the specific research bottlenecks and training gaps** at home institutions and in DECs. This maybe an underused opportunity to gain information that can be incorporated into the programme.

RECOMMENDATION: Increase **hands-on training**. Although the programme already offers a lot of hands-on work as compared to theoretical study, participants reported that more would be useful.

In particular, providing additional training in grant-seeking and grant-writing skills could boost the number of grants obtained by fellow in the post-programme period.

4.3 CDF programme ability to increase DEC institutional capacity for PD/CT Research

Effectiveness of the CDF programme with regard to mid-term impact on DEC institutions was assessed by reviewing the reintegration of fellows post-training, and the impact of the fellow's return on research and collaborations at the home institution.

Reintegration of the fellows after their training

The smooth reintegration of fellows is necessary in order for DEC-based home institutions to reap the benefits of the CDF programme. This is an important aspect of the programme in itself as well as an important step along the path that leads to impact at the broader DEC level.

Fellows are required to remain in their home region for a least one year following the end of the CDF programme. We have known of fellows being placed in companies and organisations in their region and keeping contact with their home institutions. TDR reports that one fellow from China who spent her training period at Novartis in Basel got a one-year position at Novartis Shanghai after coming back to her home institution with a preceptorship (1-2 days per week) at her home institution. Two fellows from the African region are working at EDCTP and AMANET. The number of surveyed fellows still working at their pre-programme home institution was 16 (76% of respondents).

When fellows were asked about the ease of reintegrating into their home institutions, their responses ran the gamut from "very easy" to "very hard." Thirty-eight percent (8) of fellows said that their reintegration was "problematic." Three fellows reported that problems with re-entry were not resolved. One fellow commented that "TDR should make sure home institutions understand what they agreed to, and prepare the ground together with the home institution for the fellow's return."

All fellows surveyed had been able to take leave from their home institution for the entire duration of the CDF programme and about half of them reported that their salary was paid during the training period (48%). According to their agreement with the CDF programme, home institutions must reintegrate their fellow at their pre-programme level, although not necessarily on the pre-programme project. Fourteen percent of the fellows surveyed had been replaced in their absence. In two cases one or more projects were halted or delayed due to the fellow's absence.

*"**The problem in this training is the home institution**. Before departure, everything is clear <and> all the papers are signed, but when you return … things have changed. We do not know if the institution was ready to do clinical trials. **This programme was not only for my home institution, it was also to my country, if my home institution does not want to benefit from this programme, another institution in my country will benefit from it**." – Former CDF fellow*

RECOMMENDATION: **Improve engagement of home institutions from the beginning, ensuring that they benefit fully from their fellows' newly gained skills and competencies following reintegration.**

The establishment of reintegration protocols could help home institutions to take advantage of their fellows' abilities at the institutional level. Involvement with the CDF programme from the beginning can facilitate the necessary information and level of engagement to ensure a smooth and effective reintegration.

Impact on the home institution and country environment

*"My participation to the fellowship was the capacity building component of a larger program to undertake clinical development of malaria vaccines. **I could greatly contribute to implement this program in my home institution thanks to the expertise acquired during the fellowship**. Preparation and long term vision is key to successful integration of trainees." – Former CDF fellow*

Although bolstered DEC institutional capacity is one of the principal stated goals of the CDF programme, it is complicated to assess, given the lack of available baseline data, on the status and needs of home institution prior to CDF programme participation. The limited involvement of home institutions in the programme itself and challenges with follow-up after the return of the CDF fellow to his or her home institution compound the difficulty. More than 47% (10) of the fellows that participated in the survey think the programme was able to address the needs of their home institutions well. Sixty-two percent (13) think that the capacity of their home institution was "increased" or "greatly increased" to undertake product development or clinical trials since their return. Of the three home institutions that participated in the survey, one saw capacity as "greatly increased", one as "increased" and one as "neither increased nor decreased".

Some fellows noted positive experiences:

*"Upon return home, I discussed with my boss who was very happy about my return, and **we decided to undertake modernization of our laboratory, and so we enrolled into an accreditation programme with the CDC**, so that we can have the capacity to conduct clinical trials, and we have been doing very well in this program. **We also developed some partnerships that permitted us to acquire state of the art equipment for research** in the domain of tuberculosis which is my primary interest. We have constituted cohorts of patients*

readily available for research in the domain of TB/HIV. We have set up an IRB/IEC for the hospital and we are reviewing protocols for research and I happen to be the president of this review board." – Former CDF fellow

While others pointed out challenges:

*"The programme is very relevant in developing individual capacity of the trainee. **However the trainee is supposed to develop within a research institution, which unfortunately is not a recipient of any support in capacity development.** The trainees thus get underutilised once they returned home. **I have been able to help in strengthening my institutions capability, but we are still not at the level of implementing GCP-compliant trials**" – Former CDF fellow*

The impact of fellows on their home institution after completion can be multiplied by their participation in training activities. CDF fellows surveyed have participated in a range of different training activities. In order to adequately measure impact it would be necessary to acquire baseline data that would allow estimation of the translation of the fellow's new skills to degrees of change at the institutional and country level.

Participation in Training Activities Post-CDF Programme (Fellows' Survey)		
**Organised or taught other scientific courses**	13	**62%**
**Organisation, planning or teaching of GCP or GLP courses for home institution staff**	12	**57%**
**Teaching Master's degree or PhD students**	10	**47,5%**
**Supervised or co-supervised a Master's degree student**	10	**47,5%**

RECOMMENDATION: **Create the necessary conditions to support the home institution** in providing a working environment and projects that encourage the continuing professional development of the fellow and institutional capacity building based on the fellow's new skills. This will remain a difficult task, but it is one that could be addressed through several areas of action:

- Focus on home institutions that are willing and able to support institutional and individual capacity building and that include these issues in their human resources development plan.
- Include home institution leadership in the process from the beginning and be open about problems that are likely to appear in the course of the programme so that solutions can be discussed in advance.
- Identify a mentor for each fellow at his or her home institution and support that the mentor is in regular interaction with the fellow.
- Undertake field visits by CDF management to home institutions following the selection of the fellow. Include home institution representatives in twice-yearly alumni meetings so they have a forum in which to report on their activities, raise their issues and challenges and have the chance to learn from one another's experiences. Include such activities in the budget plan.

Interaction of home institutions with the international scientific community

The programme increases the exposure of fellows to the international scientific community and this has, in turn, led to increased contact between home institutions and the international scientific community. After returning to their home institution, 66.5% of fellows (14) participated in congresses or meetings related to PD/CT at the international level. Fifty-seven percent of the fellows established one (7) or more (5) additional national/international research collaborations during their training and 81% established a collaboration following their training. Here, we see room for improving the interaction of the fellow with other institutions during the training.

RECOMMENDATION: Provide more **exposure and training related to international collaborations** and institutional alliances and partnerships as well as networking.

Collaborations help to alleviate scientific isolation and amplify the impact of the fellow's new skills to impact scientific work at the institutional and even the country level. Exposure to collaborations and skills that facilitate collaboration should figure into CDF training prominently.

4.4 Online resources and tools

The effectiveness of the CDF programme's online resources and tools was measured in terms of design, timely launch, and maintenance, as well as use of these tools by programme stakeholders.

The TDR Fellows website was created to provide a dedicated platform for fellows during the programme and an online community for programme alumni. In addition, an innovative initiative, the web-based Continuing Professional Development (CPD) tool, was developed within the scope of the CDF programme. This initiative has led to the creation of a platform that is used internationally by users from a range of disciplines. The re-named "Professional Membership Scheme (PMS)" is located within the Global Health Trials (www.GlobalhealthTrials.org) area of the Global Health Network (GHN), a group of associated websites that also includes the TDR Fellows site. It contains a wealth of resources including tools, templates, and contacts; and provides extended networking opportunities.

The TDR Fellows Website

The current TDR Fellows website (www.TDRfellows.org) was designed and launched with Phase II of the programme and has been functioning as the main site for the programme since then. Its development was outsourced to the *University of Oxford CPD operational management team*, through a BMGF grant (*CDF grant 51644*).

The site, which includes eight pages (and subpages), is password protected and is exclusively for the use of past and current CDF fellows, CDF candidates, and CDF partners. Registration is straightforward and communication with the technical support team is easy. Information and resources available on the site include: community discussion boards, programme news, links, events, congresses and meetings, online workspaces, and a section dedicated to CDF programme progress reports. A user directory with contact information is also available.

"This web space is for past, current and future members of the TDR career development fellowship scheme. The aim is to provide a facility where fellows can exchange experiences, ideas and gain support

before during and after their fellowship placement. Membership is therefore restricted for the rest of this site to past, present and future fellows and the wider members of this programme, including supervisors and sponsors."

– TDR Fellows website (http://tdrfellows.tghn.org/)

TDR Fellows website registration is mandatory for fellows and has been completed by all but two (93%). In total, 58 users are registered, including host company mentors, TDR management and the editorial team.

TDR Fellows Website traffic	
Total registered users	58
Registered fellows	25
Average monthly unique visits	76*
Average monthly views of CPD pages	248*

* Average taken from the period mid October 2012 to mid June 2013

Fellows reported that among the most important aspects of the website were: networking (64%), retrieving programme information (64%), searching for advanced training options (36%), looking for network possibilities with other institutions/companies (36%), reading sign-posts (29%), and uploading qualitative reports (29%). One fellow reported using the site to seek mentoring.

Fellows' Use of TDR Fellows Web Resources (*survey data*)		
Have seen the alumni networking tool	18	86%
Use the Website	14	67%

Mandatory fellow reports are submitted online. They can be found in CDF programme fellows' newsletters, posted on the website. The full documents are found under an own section "members and progress reports". One home institution mentor reported making use of the site to read his fellow's reports and to look for new training and network possibilities, alumni networking and even mentoring his/her fellow. None of the host companies that participated in the survey had used the website.

Since the number of CDF participants was limited during the early stages of the programme, the TDR Fellows website community has been too small to be as highly-active and vibrant as it could be with a larger pool of users. The development of the online community has also been limited by the fact that only a small proportion of users actively contribute to the site, which is a common experience in the development of interactive websites. These conditions will naturally change as increasing numbers of CDF participants register on the site and a critical mass of users is built. For this reasons, we think a wise decision, to place the CPD tool within the wider context of the Global Health Trials website, was made at the beginning of Phase II.

Of the 27 CDF programme fellows from Phases I and II, all nine from Phase I and 16 out of 18 from Phase II are registered as members in the TDR Fellows website. All new fellows from latest round (13 accepted) are already registered as members.

Six fellows from Phase II presented their 6-month progress reports online and five presented their 12-month progress reports online. There were no re-entry reports available online from Phase II.

Under the "Community" section, only two fellows from Phase II have been active, along with a web administrator. In the subsection "groups" there are eight posts, the first from two years ago and the last from two weeks before the last analysis in June 2013. On the blog we only saw activity from web administrators.

RECOMMENDATIONS: **Create incentives to increase use of the PMS site and to increase exchange and participation on the CDF website.**

Currently, website use by CDF programme alumni is limited to fellows. However, in focused interviews with home and host mentors, we heard several times that an online networking platform would be useful. Use of the website and communication between home and host institutions online would also make the site more valuable for fellows as well.

- Be creative in **challenging and stimulating representatives** of the home and host institutions, i.e. reminders for the download sections of the reports, and follow-up of the discussions of other CDF programme participants. The assignment of focal persons at the home institutions will reinforce the use of the online platform by the DEC partners. Consider downloading options of fellows' reports by the mentors through the website only.

- **Organise and label site content** for more user-friendly navigation. The site could benefit from a reorganisation, and elimination of redundant information. *For example, "workspaces" is its own tab and is also found as a subheading under "community". At the same time, "community activity" can be found under "workspaces". The tab "members and progress reports" should be renamed "progress reports", and tab "re-entry form" should be renamed "re-entry reports".*

- **Communication** would be **improved** by harmonizing the three sections "community activity", "blogs" and "groups". Be clear in which sections is within the restricted or the open network space, respectively.

Future University of Oxford CPD operational management team activities already take this direction. Plans include the redevelopment of the GHN --changing current discussion groups into discussion forums -- which will be rolled out across the entire network (the constellation of sites that includes Global Health Trials and the "TDR Fellows"). Redevelopment will also include user profiles that are seamlessly linked to the PMS (there will be prompts to encourage the creation of a full user profile). This will contribute to ensuring that CDF programme participants will be integrated with the GHN.

The PMS Online Resources and Tools

As stated above, CDF programme documentation includes a TOR, "To build an on-line continuing professional development (CPD) scheme for clinical trialists working in global health". The CPD tool was intended to support the CDF programme as an online resource that would provide direction, signposting and support – a platform through which CPD points could be accrued, audited professional records could be maintained, and mentoring and support could be provided to clinical research professionals. Target users were to include trainees, peers, and mentors, and contain regular progress reviews. It is now called the Professional Membership Scheme (PMS).

The intention was to create a resource independent of the restricted TDR Fellows website so that contents would be available to a wider audience. The PMS is now available via the Global Health Trials

website (www.globalhealthtrials.org). It has developed much as described in the initial documentation. PMS users post profiles through which their development and skills acquisition is measured and tracked to capture advancement. The PMS currently has 292 members, eight of which are TDR fellows. More than 5.000 people have taken an available eLearning course. TDR fellows are able to take advantage of the discussions, advice, literature, standard operational procedures resources and the new "Site Finder" tool.

Upon registering at the TDR Fellows website, users are given an orientation by the website manager that includes information about parallel online resources and are encouraged to regularly visit the Global Health Trials website (https://globalhealthtrials.org). There is some uncertainty as to what extent the CDF programme participants are aware of the relationship between the TDR Fellows website and the PMS. It was not clear to the evaluation team since first, none of the survey respondants mentioned the PMS and only referred to the TDR Fellows website; and second, some expressed interest in more useful and updated content, which they could have had already if they had accessed the PMS and Global Health Trial pages.

Auditing and validation is performed for 15% of PMS profiles (all members are to provide references). This process is being reviewed by an independent Oversight Committee.

The CPD operational management team reports to the CDF management quarterly by teleconference and in person every two years.

RECOMMENDATION: **Be creative in regularly educating** CDF fellows and other partners/stakeholders about the online tool(s), **publicize the PMS** more widely, follow up on **accreditation**, and develop a continued **marketing strategy**.

The PMS is an excellent resource that could provide support for many more young professionals.

It may not be clear to all CDF participants that the Global Health Trials website and the PMS are available to them. The need to access these resources via two different sites may be confusing and also discourage use, especially for the host and home institution mentors. It is therefore important to be clearer about the websites, their points of access and the resources they provide for CDF programme participants. For example:

- Increase harmonisation of the two sites, i.e. consider entry to the TDR Fellows website through the Global Health Trial portal, so it is easier to understand how it fits into the network;
- Provide specific information about the nature and characteristics of the various online tools and resources (i.e., through webinars and the newsletter);
- Ensure that the TDR management team continuously raises awareness to CDF programme participants.
- Use the potential that this platform offers for continuing support to fellows and home institutions after the training period in the CDF programme.

Pursue options for PMS points to accrue and for accreditation.

Develop a marketing and communications strategy for the PMS tool.

In theory, CDF Web site registration is mandatory for fellows, although in practice not all fellows have accounts. Integrating the CPD tool into the CDF site and making use of the CPD tool mandatory for all participants (fellows, home institutions, and host companies) will facilitate a more interactive, useful and updated resource.

Use of Website for Alumni Networking

This section focuses on the *online* aspect of alumni networking only – a comprehensive assessment of the alumni network itself is found in section 4.5, “Alumni networking and support”. Although the TDR Fellows site is fully functional, and all the essential elements of the site are in place, its overall usage is limited. We consider that this is due to the limited number of CDF fellows. In this context, the main challenge is that very few host company or home institution mentors have chosen to register.

Fellows reported that they would benefit from both

- Regular use by all fellows
- More useful and updated content.

Even when outside the evaluation period, it is, however, worth noting that the website has recently seen 13 new members having signed up from the new round of fellows, bringing the total of registered fellows to 38 out of 36 which is a milestone towards a more busy alumni networking.

4.5 Alumni networking and support

Effectiveness of the alumni networking and support was assessed in terms of the post-programme contact between TDR management and CDF programme alumni, annual alumni meetings, the establishment of a follow-up grant scheme to returning fellows and the alleviation of scientific isolation.

Post-Programme Contact between TDR and CDF Alumni

Outreach and communication efforts by the TDR management team are regular and take place with past, current, and future participants (fellows, host companies, and home institutions). Contact is made primarily via e-mail and telephone. The level of contact with programme alumni varies greatly from person to person and depends primarily on personal response. Former participants that are in regular contact with the CDF management tend to be those who continue to participate in the programme, i.e. as host company mentors.

Annual CDF Alumni Meetings

To date, two alumni meetings have been held – one in 2010 and one in 2012. Meetings include presentations from WHO representatives, CDF management, and members of the programme; information about the programme, partners, funding, exchanges among past and present fellows, and opportunities for networking.

Attendance at CDF Alumni Meetings

Year	*% of former fellows*	*% of host companies*
2010	90,5 (19/21)	56 (9/16)
2012	96,3 (26/27)	69 (11/16)

As reported by fellows, common issues and concerns addressed through the meetings included:

- Continued research collaboration between alumni
- Placement of fellows at different pharmaceutical companies, problems encountered after completion of the programme and solutions

- Administrative issues
- Role of the home institutions

To our knowledge, there is no formal report or documentation generated from the alumni meetings, however, there is a document called "Alumni report" which is a list of identified problems with suggestions for solutions.

Alumni meetings have the potential to be very useful, although home institution participation has been limited. Host institutions and fellows have attended in good numbers, especially considering that hosts do so at their own time and expense.

RECOMMENDATION: **Establish a peer mentoring programme**.

Support connections between past and current fellows through a ***peer mentoring programme*** that matches current and past fellows. The scheme could be facilitated through the in-person alumni meetings, which would provide an opportunity for mentors and mentees to meet and begin their relationship.

Overlapping fellows at the same host company could improve on-going communications, create conditions for peer mentoring and ensure project continuity. This will be possible in future when host institutions continue to place fellows and would allow consecutive generations of fellows to hand over experiences and administrative support.

RECOMMENDATION: **Participation of host and home institution mentors in alumni meetings.**

In order to fully exploit the potential of programme alumni, it is important to get home institutions and host companies connected. This increases the commitment of all participants.

Note: Initially, venues for the alumni meetings were planned for countries with on-going CDF activities. However, high travel and time costs, together with visa problems, could prevent host company mentors from attending. Meetings held in Geneva have the additional advantage that all fellows know WHO/TDR premises and the management team.

RECOMMENDATION: **Publish documentation of alumni meetings (proceedings, agendas, minutes)** and make the documents easily accessible to all participants via the website. Improve website functions for reporting and make reporting on the website mandatory. Establish an e-alert every month to all registered users containing the recent updates.

Section 5: Results & Recommendations: Efficiency

Programme efficiency was only evaluated for Phase II, since the characteristics of Phase I and the procedures followed during that phase are substantially different from Phase II, to the extent that they would make an evaluation combining both imprecise. During Phase II many improvements were implemented based on lessons learned from Phase I.

5.1 Budget management

Budget management was assessed by comparing funding inputs to expenses wherever possible. Overall, budget documentation is minimal, which has impacted our ability to assess the efficiency of the programme.

Programme management budgeting

According to the programme proposal document submitted on 29 August 2008, the budget requested by the WHO-TDR for the CDF Programme (CDF-Phase II) scale-up was US$2,977,349, to be divided into three tranches (2009, 2011 and 2012). The goal was to award 35 – 40 CDF Fellowships over four years. The management and administration of the CDF programme was included within a TDR-WHO pre-existing programme of "Research Training Grants," and in principal did not require important additional staff costs. However, 0.5 FTE in clerical assistance and 0.3 FTE in project management were requested to help establish and manage the programme. Thirteen percent of the total budget covers indirect costs borne by the WHO-TDR.

An additional sum of approximately US$500,000 was granted in 2012 for the continuation of the programme until the end of that year. We understand from the agreement with BMGF that this extension will in fact support the programme until the end of 2014.

Budgeting per fellow

Of the total number of budgeted fellowships (35-40), twenty-seven were awarded during Phases I (nine) and II (eighteen) of the CDF programme. During Phase II, the following fellowships were offered: six fellowships in the first call (2008), five fellowships in the second call (February 2010), and seven fellowships in the third call (August 2010). In addition, 17 fellowships were offered (13 of which were awarded) in the fourth and current call (2012). The budget for each fellow, including stipend, educational allowance, travel, health insurance and meetings was approximately US$ 65,000. The BMGF has been flexible and has allowed for natural differences in per-fellow budget, based on home country and host country.

According to TDR, at the end of 2012, US$1,057,566 had been spent for fellowship placement, out of a total US$1,324,642 spent for programme activities (including the cost of alumni meetings and the website). For the same period, US$543,152 were reported as staff cost (0.5 FTE clerical and 0.3 FTE project management) and US$ 242,813 were reported as indirect costs (or programme support costs).

Up to this point, TDR management and BMGF have employed a very flexible system for making changes to the budget. In the view of this evaluation, this has been a positive tool to allow the programme to grow and develop without unnecessary contraints.

RECOMMENDATION: Although the evaluation finds that the current flexible budget system is positive for the CDF programme, a **clear budget report reflecting the changes made over the course of the year** should be prepared annually, revising estimates from the previous years and attributing expenses by category including average cost per fellow for each year.

RECOMMENDATION: Add a **budget category to support home institution involvement.**

5.2 Calls, applications, selection, and milestones

The evaluation looked at the efficiency of the call for fellows, application and selection processes and milestones based on how well pre-established deadlines were met. Overall, we saw delays in all recruiting rounds, but a generally satisfactory meeting of deadlines that appears to be improving over time.

Call, application, and selection processes

Most fellows who responded to the survey thought that the call was issued in a timely fashion (91% either "agreed" or "strongly agreed" with that statement). Ninety-five percent reported that the application instructions were easy to follow and application requirements were made clear (95%). Slightly fewer (77%) answered that the selection process was transparent and that notification of selection was made early enough for necessary planning. Ninety percent of fellows thought the selection process was carried out well. A more complete evaluation of the selection process can be contemplated in the next round by gathering feedback and data from candidates who are not ultimately selected as CDF fellows.

In contrast, host companies who answered the survey were less positive about this process. Only 44% thought the call was issued in a timely fashion. Sixty-six percent reported that application instructions were easy to follow and 78% that application requirements were made clear. Sixty-seven percent of responding host companies thought that the selection process was transparent and that notification of selection was made early enough for necessary planning. Host companies had a similiarly lukewarm overall impression of the selection process. In principal, the selection process was meant to be participative and to include the CDF management team, host companies, and fellow candidates. A majority of host companies (67%) agreed that it met this requirement.

Each recruiting round has seen more applicants than positions offered (see table in section 4.1), which has allowed for shortlisting. At the same time, in each round some positions offered by host companies have gone unfilled due to lack of an appropriately qualified candidate. In these cases CDF management has opted for quality rather than quantity and has left the positions empty rather than selected a fellow who is not an optimal match for the needs of the host. Fellow nominees are usually chosen by their home institution on the basis of their individual candidacy, rather than based on a comprehensive institutional or national capacity-building strategy (see section 4.1).

RECOMMENDATION: Continuing growing the programme, with a **continued focus on quality.**

Up to this point, CDF has managed to grow while maintaining a focus on the quality of fellows and the quality of host-fellow matches. There were cases where placements would have been open but the candidates' and host institution's profiles didn't match. In these cases, quality of host-fellow matches was followed as a decision point superior to placing as many as possible fellows. This is a good pattern to follow. Upon expansion of the programme in number of participants and partners, we advise to adhere to the fitting quality before quantity paradigm.

RECOMMENDATION: **Document and benchmark the selection process.**

Documentation will allow the CDF to set new recruitment goals (number of contacts made, number of applications received per available position, number of application that meet the requirements, marketing channels being used…) for each round and to accurately evaluate the previous round of admissions based on past performance.

RECOMMENDATION: **Think creatively about programme partners.***

To maintain growth, CDF should invest in incentivizing current partners to continue their association with the programme and look to "non-traditional" hosts as well, such as PDPs. Consider opening the training scope to include more topics relevant to applied research (i.e. operational research, implementation science, pharmacovigilance…).

**In the fourth round of recruiting (not included in this evaluation) PDPs have been already included as hosts.*

5.3 Addressing issues and challenges

Challenges naturally arise in the implementation of any programme. Here, we look at the ability of CDF management to handle issues and the efficiency with which they and other stakeholders managed challenges that were identified during the alumni meeting in 2010 and 2012.

Challenge	Response
**Delay in placement of fellow at host company**	According to the information from several programme participants, these issues have been taken care of rapidly by CDF management, drawing on extensive knowledge of offices and procedures. Although, of course, not all problems could be solved, such as country-specific challenges like visas and contracts.
**Administrative status of fellow at host company**	Administrative status issues are now clarified between CDF management and host companies when the fellow position is being created.
**Integration in host countries and host company working culture**	Some fellows have experienced challenges integrating into their new working environment. Efforts are being made by both host company mentors and CDF management team to resolve this issue. Mentoring is key here and mentors, available by phone and e-mail, are identified at host companies. An overlapping schedule of fellows has not yet been possible. In the future, this would allow

	for the possibility of peer learning and mentoring.
**Reintegration of fellows at home institutions**	Measures to address issues rising during the reintegration of fellows into their home institutions have been extensively discussed within the CDF management team (see previous comments). Obstacles to the smooth reintegration of fellows were identified and roles and responsibilities are now being clearly defined. The selection process now focuses even more on the long-term needs of home institutions, a re-entry grant scheme is being considered and more involvement from home institutions is seen as important.

Issues and challenges were identified during the initial rounds of Phase II and were handled with in an efficient manner even when not all of them could be solved.

RECOMMENDATION: **Document problems and create action points.**

More extensive documentation of problems that have arisen will allow for sticking points to be analysed and for solutions to be proposed and implemented. Include suggestions for action points including answers on whom, how, and until what point issues should be addressed.

5.4 Oversight and management of the CDF programme

Athough the overall quality of the CDF programme does speak to the quality of its management, in a sense it is difficult to evaluate CDF programme management because of a lack of documentation (as raised earlier in this report).

The Oversight Committee

The TDR-CDF Programme Oversight Committee includes the TDR Training Committee and the TDR Strategic and Technical Advisory Committee (STAC). TDR reports with results of the CDF programme are included in annual reports of the Research Capability Strengthening and Knowledge Management team (RCS/KM) and are presented to the STAC and the Joint Coordinating Board for comments. The Training Committee and STAC receive annual reports on CDF activities, which we also have not seen.

The secretariat of the CDF programme or the “CDF management team,” consists of 0.8 FTEs per year, including the coordinator of the CDF programme. The team meets informally on a day-to day basis and holds regular meetings to address operational issues. Regular reports to update activities are delivered to the BMGF by e-mail.

CDF Management Team	
**Manager**	0.3 FTE
**Technical officer**	0.3 FTE
**Administrative assistant**	0.2 FTE

RECOMMENDATION: **File reports** made to BMGF and **document Oversight Committee** meetings.

5.5 Partner's roles and responsibilities

Roles and responsibilities are clearly defined for **CDF management, host companies** and for **fellows** in:

- "TDR's Clinical Research CDF programme" and
- Clinical Research Career Development Fellowships – General Information and Financial Provisions"

Participant	Responsibilities	Evaluation
**CDF Management**	• Coordination of the fellows' recruitment and placement;<br>• Management of the administrative (including visa entry applications) and financial aspects;<br>• Oversight of the individual training plan and activities to ensure their compliance with the programme objectives;<br>• Organisation and management of an alumni network for past and current fellows;<br>• Monitoring and reviewing the on-going programme.	Well executed
**Host Companies**	• Selection of suitable candidates in collaboration with TDR;<br>• Supporting TDR and the fellow(s) in administrative (including visa entry applications) and logistic aspects linked to the settlement in the host country;<br>• Provide the fellow(s) with opportunities and activities relating to clinical research for product development;<br>• Provide an environment that supports the fellow(s) towards achieving the objectives of the CDF programme (including engaging of the fellow in host institution team activities in a clinical trial)	Well executed
**Fellows**	• Full participation in the pre-agreed clinical research activities of the host company;<br>• Submit a progress report after 6 months and a final report at the end of the placement (including content, skills acquired and experience gained);<br>• Return to their home institutions at the end of the placement to work in the field of their training;<br>• Submit a follow-up report after additional 12 months (including progress and pursued activities in line with the CDF programme and other activities and contacts).	Individual assessment
**Home institutions**	• Ensuring the training will enhance the fellows capacity;<br>• Agree to grant leave of absence and guarantee the fellow a post upon return which is relevant to the fellow's training and will make use of the knowledge and experience gained.	Not well defined

*"Since **the home institution was not updated and included** during the one year programme, it was hard for them to understand how to proceed upon return." – Former CDF fellow*

Roles and responsibilities are not well-defined for home institutions. Surveyed fellows, however, reported that they were clear on roles and responsibilities for *themselves* (95%), *CDF management* (90%), and *host companies* (86%). Just 43% felt that the role for their home institution was clear. A similar percentage of fellows responded that each of the roles was realistic and that they were well carried out.

*"My institution has been **quite supportive considering the limited resources** available for research." – Former CDF fellow*

Surveyed host companies were clear on roles and responsibilities for *fellows* (78%) and *themselves* (89%), but lacked clarity on *CDF management* (33%) and *home institutions* (22%). Hosts responded similarly regarding whether roles and responsibilities were realistic and well carried out. Eighty-nine percent were satisfied with the contribution of their fellow.

*"One gets the impression that **the home institution was not really aware of its role**, was not really involved in the selection process and hence missed out opportunities." – Host company mentor*

In some cases the unclear role of the home institution may have been further complicated by fellows who did not stay in touch or regularly report back, leaving the home institution without necessary information to carry out is role, especially with regards to reintegration.

RECOMMENDATION: **Refine roles and responsibilities of home institutions**, and enhance their visibility.

- Roles and responsibilites should appear in Letter of Award.
- Fellows should be obliged to ensure ongoing communication with their home institutions.
- It is likely that both a clear definition and communication of the roles and responsibilities will be essential to an increased understanding of the home institutions' important place and their active participation in the CDF programme.
- Increase home institutions' engagement in the programme by defining additional roles and responsibilities.

5.6 Interrelations

Participant relationships, communication, accessibility, and mutual trust

Communication between CDF management, host company mentors, and fellows is generally quite efficient.

Fellows reported that CDF management team was easily accessible when approached. All administrative issues were addressed and, where possible, resolved.

With few exceptions, host companies were able to integrate fellows into their new working environment. With one exception, host companies assigned each fellow a mentor, and fellows were in regular (more than once a week) contact with that mentor. According to the fellow surveyed, in two cases mentors were also in regular contact with the home institution.

In general, communication with home institutions was more difficult, although it varied widely from case to case. While a few fellows had regular and productive communication with their home institution during the CDF year, for many fellows the fellowship period was effectively meant quitting and then re-taking a job – with little communication in between.

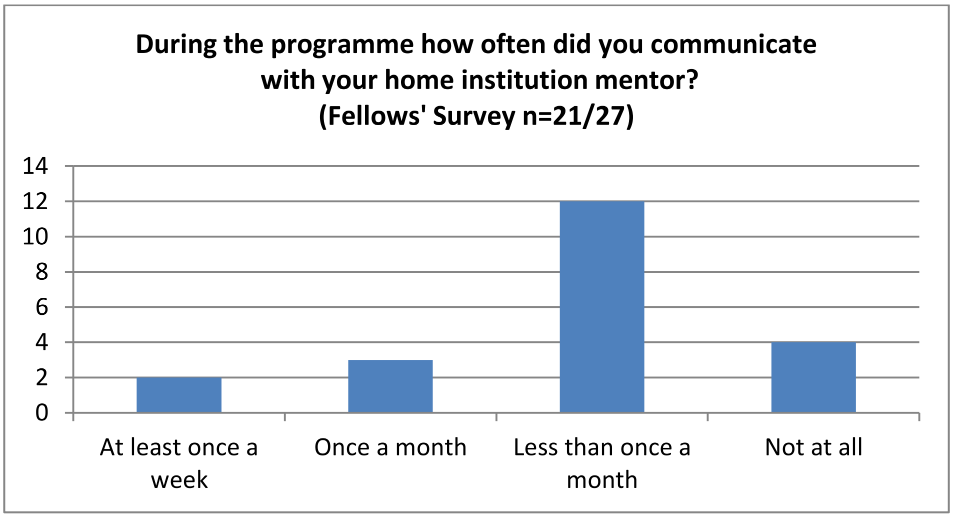

Mentoring during the CDF placement

While home institutions were only marginally involved in project design, supervision of fellows, project development and implementation, the host companies took their task of training very seriously. Host companies assigned mentors, set training objectives, engaged in continuous communication and follow-up activities. Fellows' administrative issues were addressed by CDF management and host companies. Issues were addressed as quickly as possible.

Continued association and contact of the fellows with CDF management and host companies

Communication between fellows and host companies continues beyond the fellowship period in most cases. This is also true of communication between host companies, fellows, and CDF management.

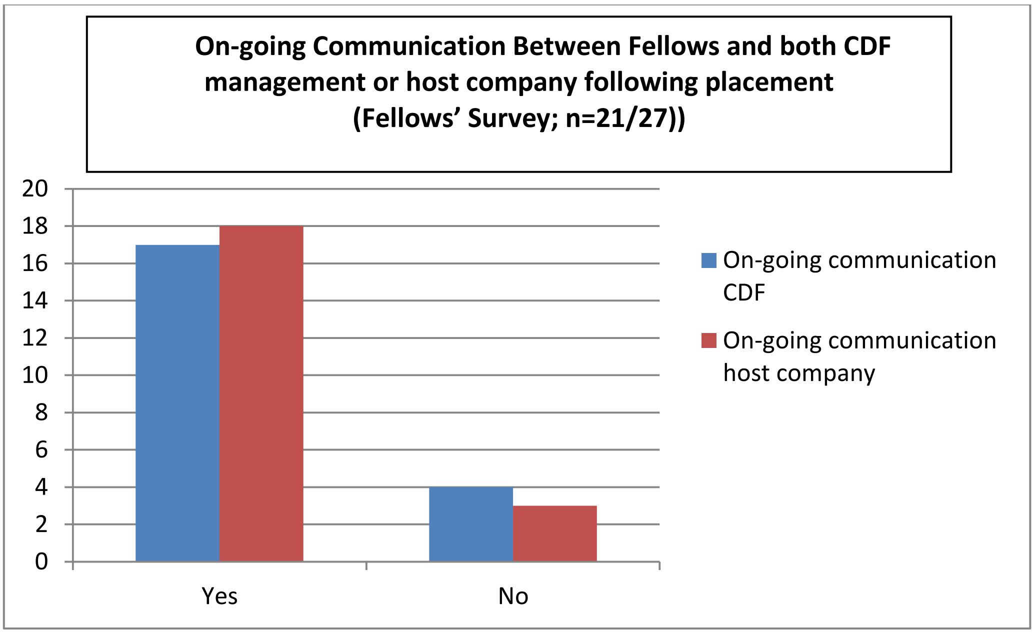

RECOMMENDATION: Continue communication between fellows, CDF management, and host institutions.

Support communication between fellows and either CDF management and host institution by making use of the available tools (website, PMS, alumni meetings…) or any other new initiative.

Facilitate tools and promote comunication of fellows with their home institutions during the training period by defining better home institutions' roles.

5.7 Reporting

Reporting to BMGF

Programme progress reports by CDF management were delivered to BMGF in November 2010 (for the period November 2009 to July 2010) and in October 2011 (for the period August 2010 to September 2011). The report initially planned for fall 2012 was agreed to be postponed since the funding period was prolonged. As a consequence the new cohort of fellows placed in spring 2013 should be part of the next progress report. Plans are communicated to BMGF by telephone and through quarterly e-mails.

Fellow progress reports

Progress reports by CDF fellows, to be delivered to the host company and to CDF management, are due six months after placement, 12 months after placement, and one year after return to the home institution. On the CDF programme website, seven fellows have delivered their six month reports and five fellows have posted their final report. No one post-programme report has been published as of this time. TDR has stated that all 6 month reports and all (but one) 12 months reports have been received by the secretariat. Only one out of four one year post-return reports has been delivered.

RECOMMENDATION: **Emphasize reporting deadlines for fellows** and post all fellow reports online.

It will help to make acknowledgement of the receipt of reports mandatory for all participants (home institutions, host companies, CDF management).

Section 6: Results & Recommendations: Impact and Sustainability

In this section, the evaluation considers two key elements: impact in the mid-to-long term, and the sustainability of the programme and its activities.

6.1 Impact

"The CDF has been helpful in the development of my career. I am currently the administrator of the IRB ensuring training and ethical conduct of studies including clinical trials in my institution. With my leadership ***we were able to get funding for strengthening the IRB. My teaching has certainly improved and I have changed ranks from an assistant lecturer to a senior lecturer****." – Former CDF fellow*

The impact of the programme has different dimensions for mid-to-long term.

Short-term impact on fellows and their research environment

It is clear from the feedback of all participants (fellows, host companies and a few home institutions) that at an individual level, learning objectives have been reached and that the overall level of satisfaction is very high (see section 4.2).

We note that as programme documentation references the "establishment of re-entry grant scheme" is still under development.

"Re-entry grants and home institution strengthening ought to be a priority *if the fellow is expected to effectively put to use the skills acquired during the training." – Former CDF Fellow*

Alleviation of scientific isolation

Since I returned home… I am fully involved in so many projects with partner institutions. ***I do research with some Western universities working with us****. I have learned a lot since I returned and* ***I am trying to help young colleagues*** *to do research so that we can constitute a team. – Former CDF Fellow*

One of the long-term purposes of alumni networking is the alleviation of scientific isolation, through the creation of an international community of research scientists available to one another for consultation, professional support, and possible cooperation.

Fifty-eight per cent of the fellows reported that alumni activities and meetings do help alleviate scientific isolation. All respondents had participated in at least one alumni meeting, and 86% of them rated those meetings either "useful" or "very useful."

Mid-to-long term impact on fellows and their research environment

We were initially involved in implementing a research study; unfortunately ***this initiative fell for multiple reasons including the lack of a clear national framework for clinical trials, ever changing regulations and a limited number of trained staff*** *at the institution. – Former CDF Fellow*

It is difficult to estimate the impact of the programme at the institutional and country levels. The response of home institutions has been too low to gauge the implications of the fellows' new and improved

competencies and skills for their home institutions. This is another area that could benefit from more involvement and a more clearly drawn role for home institutions participating in the CDF programme, as discussed earlier in their report, as well as of a good baseline definition of “research bottlenecks” and “training gaps”.

*“The CDF programme is a very interesting programme … With this programme I (have) specialized in clinical research … I have learnt a lot during this training **and I will be able to set up an investigational clinical site and conduct clinical trials in my country where clinical research is still a new concept**.” – Former CDF Fellow*

Such involvement could assure the translation of the individual capacity into institutional capacity and, consequently, the assessment of impact at the institutional level, and even at the national and regional level, would beome more manageable. Of course, a precondition of this involvement would be the existence of adequate scientific and regulatory environments for PD/CT projects.

*“**We have had ten [fellows] and all are still in Africa**. Three are not in their host institutions now but with other research groups but all went back. One went on to do a PhD and now works for a pharmaceutical company.” – Host Institution*

*“It is hard to say over a 3 year time frame which activities the fellow will be involved in. **On his initial return, it seemed as if his institution is not planning to take advantage of his new / improved skill set**. **By selecting a junior fellow, he does not have the seniority on his return to be a driver of activities or guide his institution**. Such roles seem to depend more on age / seniority rather than experience or competence.” – Home institution*

RECOMMENDATION: Seek funding for and **implement a follow-up grant scheme**.

A follow-up grant scheme could cover infrastructure and project expenses for projects in accordance with the learning goals during the CDF placement. A programme of this kind would serve help smooth fellows’ return to their home institutions and secure continued professional development for CDF alumni. Obviously, this will require funding for projects that meaningfully link new work to knowledge and skills acquired during the CDF training period. Ideally, this type of scheme will also strengthen the link between home institutions and host companies. In the next round of funding budgeting should include this scheme.

6.2 Programme Sustainability

Sustainability is essential to achieve the mission of the CDF programme, as well as to assure the long-term goal of translating individual capacity into institutional capacity. Some stakeholders have suggested that this particular goal would be best achieved through a combination of actions designed to strengthen scientific capacity and the regulatory environment for PD/CT in DEC countries and regions.

As previously mentioned, the clear definition of roles and responsibilities for each CDF programme participant, whether TDR-CDF management, fellow, host company or home institution is fundamental to the success and sustainability of the programme and is an area for improvment in subsequent calls or programme phases, particularly in reference to home institutions.

Another issue of concern for CDF programme sustainability was raised by several fellows, and is the need for a means of formal academic recognition for CDF training, which would significantly increase its value in the context of continuous professional education or postgraduate education (including Master's degree and doctoral programmes).

Follow-up of TDR-CDF programme management

Two-thirds of the CDF fellows reported that CDF programme management had followed-up with them post-programme.

On-going communication & continuation of collaborations and research projects

Although most host companies contacted (78%) kept up communication with fellows after their return to their home institution, they reported no continuity in the collaboration with the home institutions.

Satisfaction with the CDF programme

The fellows surveyed all said that they would recommend the CDF programme to colleagues, reflecting a generally high level of satisfaction with the experience.

Two-thirds of participating host companies said that they would continue to offer positions for CDF fellows (one third answered "do not know"). In interviews, host companies and the TDR manangement team confirmed a high level of satisfaction with the programme. Although in some cases problems had arisen for host companies, they had been satisfactorily resolved in collaboration with the TDR management team. The relationship here, between hosts and TDR-CDF management, is critical as the sustainability of the programme resides very much in the ability of the CDF programme to maintain and grow a pool of collaborating host companies.

Although fewer home institutions participated in this evaluation, those contacted were prepared to present new candidates for CDF training.

Long-term financial support

The CDF programme is relatively costly to run and of course its sustainability is very much dependent on the mid-to-long term financial support available. Two models could be considered to guarantee the continuation of the programme in the future.

The "single-funder model" is the current and historical approach and could continue to work if the BMGF is prepared to keep supporting the CDF programme into the future. The single funder model has the advantage making donor relations and management easy and flexible. TDR has brought its expertise in research capacity development and grant management to this relationship. On the other hand, the drawback is that continuity and sustainability of the programme becomes entirely subject to the priorities and agenda of one organisation, priorities that naturally could change over time.

A "multi-funder model" implies the involvement of new funding partners. An increase in the number of funders/funding would permit the programme to grow and extend to more offers (given that host companies had places available). Extra funding could also be channelled into supporting complementary programmes, like re-entry grants, joint projects funding, etc., ideas that have been raised by former CDF fellows.

An alternative approach raised with regards to funding is to ask host companies and home institutions to shoulder some of the costs of the programme. Of course, it is important to consider that, in one way or another, both hosts and home institutions already assume some of the costs inherent in welcoming a new staff member to the lab or replacing a staff member who is away for a year, leaving his or her position vacant. Some host companies already contribute with accomodation, additional training and conference attendance costs. None of the home institutions who responded to the survey thought it would be reasonable for their institutions to assume more costs and just one out of the 9 responding host companies was in favour of such an approach.

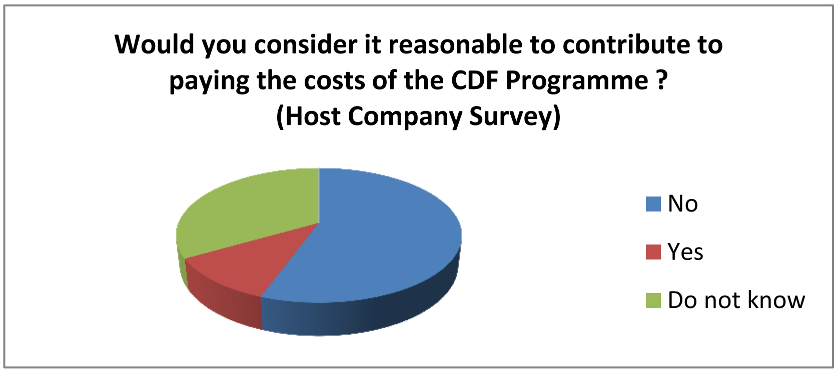

RECOMMENDATION: **Strengthen focus on institutional continuity** to ensure on-going collaboration between CDF, host, and home institutions. Such interactions will result in long-term individual capacity development as well as fellows sharing overlapping CDF training schedules, leading to more peer mentoring and mutual learning.

RECOMMENDATION: Establish a **long-term funding strategy** that takes the pluses and minuses of both the single-funder and multi-funder approaches into account.

RECOMMENDATION: **Consider seeking an academic partner** or partners so that CDF fellows may receive formal academic credit for their work.

6.3 Gender and Geographical Distribution

Gender Balance

Thus far, CDF fellows have been predominantly male (more than 80%). In future calls it would be good to encourage qualified female candidates to enter the selection process to move towards balance here. In the latest round (not included in this evaluation) gender balance is reported at 50%/50%.

CDF Fellow Gender Balance	Male
Phase I	88.8% (8/9)
Phase II	83.3% (15/18)

Geographical Balance

To date, CDF fellows have come from 16 different countries in Africa: Benin, Botswana, Burkina Faso, Cameroon, D.R. Congo, Ethiopia, Ghana, Kenya, Mali, Nigeria, Tanzania, Uganda; Asia: China and Vietnam; and Latin America: Colombia and Perú. The 14 host companies were based in Europe, the USA, and Singapore.

As we can see, the programme has mostly included candidates from African countries, with a minority of participants coming from Asia and Latin America. This geographical distribution could also be balanced in future calls, although it is evident that many African research institutions require capacity-building for PD/CT and can therefore make good use of this opportunity. According to the CDF coordinator they opted for a geographical concentration rather than for a wider geographical approach.

Section 7: Unintended Outcomes

We found several positive, unintended outcomes of the CDF programme.

First, we emphasize that the PMS on the Global Health Trials website is excellent. Initially created as a supporting online resource for the CDF programme community, it has grown well beyond into an interdisciplinary tool that is used internationally within the GHN to the benefit of a wide audience. We encourage the programme to spend some time and attention making this resource more used, better known, and available to more young professionals.

Second, TDR shared with us that the EDCTP, inspired by the CDF, has created a similar programme. This is evidence of CDF's positive effect on another organisation in their development of international capacity building. Rather than view this as a threat or as a competition, CDF management views this as an opportunity for collaboration and mutual learning. We agree, and encourage CDF to follow up and create synergies.

Third, fellows have expressed an interest in receiving academic credit for the programme. This opens up the possibility of a fruitful collaboration with an academic partner. We hope that this is something the CDF management will explore in the near future.

Finally, although the CDF programme is administered by the TDR unit tasked with individual capacity building, it has greatly contributed to strengthening the ties to and between institutions and thus reinforces institutional capacity building.

Annexes

Annex A: Table of CDF Fellows Rounds I - III

CDF Fellow	Home institution, country	Host company, country	Disease<br>Topic
**Abdullahi Ahmad**	Primary Health Care<br>Ministry of Health, Nigeria	GlaxoSmithKline Biologicals, Belgium	Malaria<br><br>Double blind (observer-blind), randomized, controlled multi-centre study to evaluate, in infants and children, the efficacy of the RTS,S/AS01e candidate vaccine against malaria disease caused by P. falciparum infection, across diverse malaria transmission settings in Africa (phase III)
**Aurel Constant Allabi**	Drug & Pharmaceutical Services, Faculté des Sciences de la Santé de Cotonou, Benin	Tropical Medicine Novartis Pharma AG, Switzerland	Malaria<br><br>Efficacy of artemether-lumefanthrine (AL) in treatment of blood stages of Plasmodium vivax
**Julius Atashili**	Faculty of Health Sciences,University of Buea, Cameroon	Pfizer Emerging Markets, Pfizer Inc., USA	Malaria<br><br>Open Label, Randomized, Comparative Study To Evaluate Azithromycin Plus Chloroquine And Sulfadoxine Plus Pyrimethamine Combinations For Intermittent Preventive Treatment of Falciparum Malaria Infection In Pregnant Women In Africa (phase III)
**Leo Njock Ayuk**	Regional Hospital Bamenda, Cameroon	GlaxoSmithKline Biologicals, Belgium	
**Steven Baveewo**	College of Health Sciences, University of Makerere, Uganda	Pfizer Inc., Emerging Markets, USA	Malaria<br><br>An Open Label, Non Comparative Study To Evaluate Parasitological Clearance Rates And Pharmacokinetics Of Azithromycin And Chloroquine Following Administration Of A Fixed Dose Combination Of Azithromycin and Chloroquine (AZCQ) In Asymptomatic Pregnant Women With Plasmodium Falciparum Parasitemia In Sub Saharan Africa (phase III)
**Qingyan Bo**	National Clinical Trial Institution, Affiliated Hospital of Nanjing, University of TCM, China	Integrated Hospital Care, Novartis Pahram GA, Switzerland	Hepatitis C<br><br>Phase II and III clinical trilas anti-hepattis C drugs
**Roma Chilengi**	Research Laboratories, Kenya	GlaxoSmithKline Biologicals, Belgium	
**Dawit Asmamaw Ejigu**	University of Medicine, Ethiopia	GlaxoSmithKline Biologicals, Belgium	
**Wilfried Mutombo Kalonji**	Programme National de Lutte contre la Trypanosomiasis Humaine Africaine, DR Congo	Sanofi Aventis (6 months), France ; DNDi (6 months), Switzerland	
**Mahmoud Yakub Ma'aruf**	Aminu Kano Teaching Hospital, Institute of Human Virology, Nigeria	F. Hoffmann-La Roche Ltd, Global Drug Development, Switzerland	Oncology<br><br>Phase 1 clinical trial for paediatric Becacizumab
**Marie Florence Makamche**	Centre International de Référence Chantal Biya pour la recherche sur la prévention et la prise en charge du VIH/SIDA (CIRCB), Cameroon	Janssen Pharmaceutical Companies of Johnson & Johnson, Tibotec-Virco Virology, Belgium	Hepatitis C<br><br>Efficacy, safety and tolerability of TMC435 versus placebo as part of a treatment regimen including peginterferon α-2a (Pegasys®) and ribavirin (Copegus®) or peginterferon α-2b (PegIntron®) and ribavirin (Rebetol®) in treatment-naïve, genotype 1, hepatitis C-infected subjects) (phase II)
**Celine Isaack Mandara**	National Institute for Medical Research,<br>Tanzania	Sigma Tau Industrie Farmaceutiche Riunite S.p.A, Italy	Malaria<br><br>Open-Label, Pharmacokinetic, Safety and Efficacy Study of a new paediatric Eurartesim® oral formulation (two strength water dispersible tablets: 80/10 and 160/20 mg Piperaquine phosphate / Dihydroartemisinin), in infant patients with P. falciparum Malaria" (phase II)
**Michel Mandro Ndahura**	Université de Kisangani CUEB, DR Congo	Novartis Pharma AG, Switzerland	Malaria<br><br>1) An open-label, single-arm study to evaluate the efficacy, safety and PK of artemether-lumefantrine dispersible table in the treatment of acute uncomplicated Plasmodium falciparum malaria (phase III)<br><br>2) A cluster randomized, single-centre, controlled, parallel, 12-month prospective study and additional 12-month follow-up in

			Africa of malaria incidence in a community setting following systematic treatment of Plasmodium falciparum (P. falciparum) asymptomatic carriers with artemether-lumefantrine (Coartem®/Coartem® dispersible) (phase III)
**Tafireyi Marukutira**	Botswana Baylor Children's Center of Excellence, Botswana	Astellas Pharma US, Inc., USA	Fungal<br><br>Study of Micafungin, BAL4815, Fidaxomicin in children (phase III)
**Laureano Mestra**	Clinical Trial Unit, Programme for Study and Control Tropical Diseases PECET, Colombia	Eisai Inc., USA	Chagas<br><br>Clinical trial (drug)
**Holger Mayta**	Department of Microbiology School of Sciences and Philosophy, Universidad Peruana Cayetano Heredia, Peru	Merck Research Laboratories Infectious Diseases, USA	Chagas / Leishmaniasis<br><br>1)Use of Oral Posaconazole (POS) in the Treatment of Asymptomatic Chronic Chagas Disease (phase II)<br>2) Safety and tolerability of a single IV infusion of SCH708980 (0.3 mg/kg, 1.0 mg/kg, 3 mg/kg, or 10 mg/kg) over the initial 7 days and in combination with a single IV infusion of AmBisome® (10 mg/kg) on the 8th day (phase II)
**Brenda Okech**	Univeristy of Makerere, Uganda	GlaxoSmithKline Biologicals, Belgium	
**Ally Ibrahim Olotu**	Kenya Medical Research Institute-Wellcome Trust Research Programme, Kenya	GlaxoSmithKline Biologicals, Belgium	
**Glory Oluwagbenga Ogunfowokan**	National Hospital Abuja, Nigeria	EISAI Inc., USA	Chagas<br><br>A novel drug in phase clinical development for treatment of chronic indeterminate Chagas diseases
**Alex Kwame Owusu-Ofori**	Komfo Anokye Teaching Hospital, Ghana	GlaxoSmithKline Biologicals, Belgium	
**Sunny Oyakhirome**	Community Health Department, School of Medical Science, KNUST, Ghana	GlaxoSmithKline Biologicals, Belgium	
**Maame Anima Attobrah Sarfo**	Komfo Anokye Teaching Hospital, Ghana	GSK UK Infectious Diseases - DDW, United Kingdom	
**Edward Steve Smith Nunez**	Peru	Pfizer, Emerging Markets, Pfizer Inc., USA	Malaria<br><br>An Open Label, Non Comparative Study To Evaluate Parasitological Clearance Rates And Pharmacokinetics Of Azithromycin And Chloroquine Following Administration Of A Fixed Dose Combination Of Azithromycin and Chloroquine (AZCQ) In Asymptomatic Pregnant Women With Plasmodium Falciparum Parasitemia In Sub Saharan Africa (phase III)
**Eric Some**	Centre de Recherche International en Santé (CRIS), Burkina Faso	Pfizer Inc., Malaria Development Program, Emerging Markets, USA	Malaria<br><br>Open Label, Randomized, Comparative Study To Evaluate Azithromycin Plus Chloroquine And Sulfadoxine Plus Pyrimethamine Combinations For Intermittent Preventive Treatment of Falciparum Malaria Infection In Pregnant Women In Africa (phase III)
**Mahamadou Aly Thera**	University of Mali, Faculty of Medicine, Pharmacy and Dentistry (FMPOS), Department of Epidemiology of Parasitic Diseases, Mali	GlaxoSmithKline Biologicals, Belgium	
**Alfred Tiono**	Centre National de Recherche et de Formation sur le Paludisme (CNRFP),<br>Burkina Faso	GlaxoSmithKline Biologicals, Belgium	
**Quoc Dat Vu**	National Hospital for Tropical Diseases, Vietnam	Sanofi France (6 months)<br>Vaccine Clinical Department, France<br><br>Sanofi-Aventis Singapore (6 months), Singapore	Dengue<br><br>1) Study of a novel tetravalent dengue vaccine in healthy children aged 2 to 14 years in Asia (phase III)<br><br>2) Study of ChemriVax™ tetravalent dengue vaccine in healthy subjects (phase II)

Annex B: Screenshot of the CDF Fellow Website

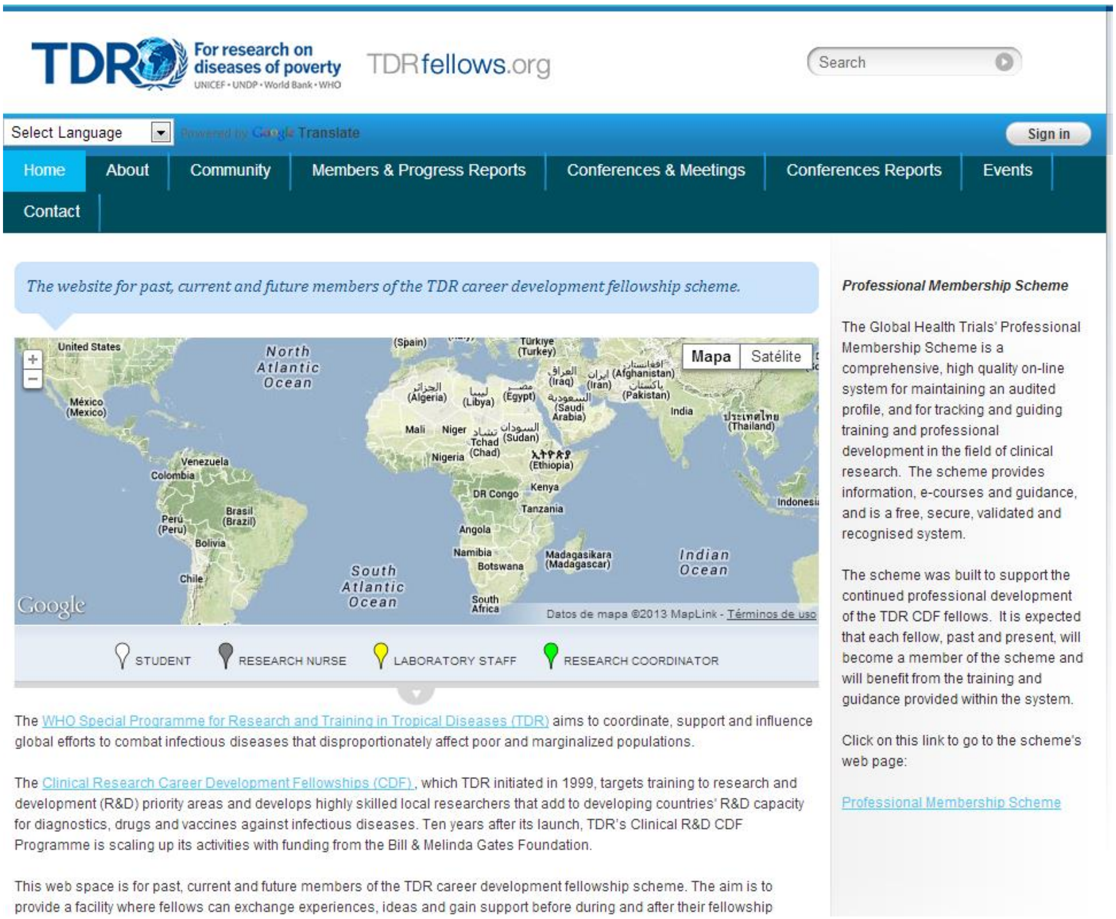

Signatures

Dr. Michael Käser, Principal Evaluator

__________________ signed in Basel ________ on 04.09.13
signature *city* *date*

Dr. Núria Casamitjana, Principal Evaluator

__________________ signed in Barcelona ________ on 0.9.09.13
signature *city* *date*
